# circlncRNAnet: an integrated web-based resource for mapping functional networks of long or circular forms of noncoding RNAs

**DOI:** 10.1093/gigascience/gix118

**Published:** 2017-11-29

**Authors:** Shao-Min Wu, Hsuan Liu, Po-Jung Huang, Ian Yi-Feng Chang, Chi-Ching Lee, Chia-Yu Yang, Wen-Sy Tsai, Bertrand Chin-Ming Tan

**Affiliations:** 1 Graduate Institute of Biomedical Sciences, College of Medicine, Chang Gung University, Guishan, Taoyuan, Taiwan; 2 Department of Cell and Molecular Biology, College of Medicine, Chang Gung University, Guishan, Taoyuan, Taiwan; 3 Molecular Medicine Research Center, Chang Gung University, Guishan, Taoyuan, Taiwan; 4 Division of Colon and Rectal Surgery, Department of Surgery, Chang Gung Memorial Hospital, Linkou, Taiwan; 5 Department of Biomedical Sciences, College of Medicine, Chang Gung University, Guishan, Taoyuan, Taiwan; 6 Genomic Medicine Research Core Laboratory, Chang Gung Memorial Hospital, Linkou, Taiwan; 7 Department of Computer Science and Information Engineering, College of Engineering, Chang Gung University, Guishan, Taoyuan, Taiwan; 8 Department of Microbiology and Immunology, College of Medicine, Chang Gung University, Guishan, Taoyuan, Taiwan; 9 Department of Neurosurgery, Linkou Medical Center, Chang Gung Memorial Hospital, Linkou, Taiwan

**Keywords:** lncRNAs, circRNAs, co-expression network, molecular interactome

## Abstract

**Background:**

Despite their lack of protein-coding potential, long noncoding RNAs (lncRNAs) and circular RNAs (circRNAs) have emerged as key determinants in gene regulation, acting to fine-tune transcriptional and signaling output. These noncoding RNA transcripts are known to affect expression of messenger RNAs (mRNAs) via epigenetic and post-transcriptional regulation. Given their widespread target spectrum, as well as extensive modes of action, a complete understanding of their biological relevance will depend on integrative analyses of systems data at various levels.

**Findings:**

While a handful of publicly available databases have been reported, existing tools do not fully capture, from a network perspective, the functional implications of lncRNAs or circRNAs of interest. Through an integrated and streamlined design, circlncRNAnet aims to broaden the understanding of ncRNA candidates by testing *in silico* several hypotheses of ncRNA-based functions, on the basis of large-scale RNA-seq data. This web server is implemented with several features that represent advances in the bioinformatics of ncRNAs: (1) a flexible framework that accepts and processes user-defined next-generation sequencing–based expression data; (2) multiple analytic modules that assign and productively assess the regulatory networks of user-selected ncRNAs by cross-referencing extensively curated databases; (3) an all-purpose, information-rich workflow design that is tailored to all types of ncRNAs. Outputs on expression profiles, co-expression networks and pathways, and molecular interactomes, are dynamically and interactively displayed according to user-defined criteria.

**Conclusions:**

In short, users may apply circlncRNAnet to obtain, in real time, multiple lines of functionally relevant information on circRNAs/lncRNAs of their interest. In summary, circlncRNAnet provides a “one-stop” resource for in-depth analyses of ncRNA biology. circlncRNAnet is freely available at http://app.cgu.edu.tw/circlnc/.

## Introduction

Only 1% of the human genome encodes proteins. In contrast, 70% to 90% of the genome can actually be transcribed at some point during development, generating a large transcriptome of noncoding RNAs (ncRNA), part of which ultimately yield definite short or long RNAs with limited protein-coding capacity [1]. In recent years, deep sequencing technologies have unraveled the noncoding constituents of the transcriptome, most notably long noncoding RNAs (lncRNAs) and circular RNAs (circRNAs). Despite the lack of protein-coding potential, these once uncharted parts have emerged as a key determinant in gene regulation, acting as critical switches that fine-tune transcriptional and signaling output [2, 3].

Distinct from small noncoding RNAs such as microRNAs and snRNAs, lncRNAs are RNA molecules with a length of more than 200 nucleotides that lack a detectable open reading frame [4]. lncRNAs are usually transcribed by RNA polymerase II and exhibit known attributes of messenger RNAs, such as post-transcriptional processing. Circular RNAs are a more recently discovered class of noncoding RNAs that are defined not by length but rather the unique structure of covalently closed circularity [5, 6]. Despite their differences in structure and biosynthesis steps, lncRNAs and circRNAs are much more common in terms of their roles and mechanisms in gene regulation, and in fact circRNAs are considered to be a class of lncRNAs by many researchers [3]. Even in the absence of protein products, these RNA molecules have been found to associate with distinct cellular compartments or components, and may act *in cis* or *trans* in target gene regulation [7–10]. At the epigenetic and transcriptional levels, lncRNAs are known to interact with transcriptional activators or repressors and consequently impact transcriptional efficiency. By binding with chromatin-modifying factors, lncRNAs could also serve as a guide or scaffold that controls the epigenetic status. At the post-transcription level, lncRNAs may bind to target RNAs and alter transcript structure, splicing pattern, and stability. Both lncRNAs and circRNAs have been found to harbor microRNA response elements (MREs) and potentially act as “miRNA sponges” that sequester these endogenous small RNAs [8, 11, 12], although the evidence for lncRNA miRNA sponges is much stronger than for circRNA sponges [13, 14]. These ncRNAs are therefore part of the competing endogenous RNA (ceRNA) network with the potential to alter miRNA-targeted mRNA expression. Another mode of regulation exerted by lncRNAs is their association with RNA-binding proteins. Similar to the ceRNA scenario, this molecular interaction may impact the localization, and thus activity, of these gene regulators. Finally, in line with their critical roles as gene regulators, both circRNAs and lncRNAs exhibit unique expression profiles in various human cancers, suggestive of a correlation with disease progression and possibly its value as a predictor of patient outcome [15–19]. Delineation of these transcriptomic networks therefore is of importance in understanding ncRNAs, and associated biological processes and may shed new light on diseases and possibly new avenues of therapeutic interventions [20–22].

**Figure 1:**
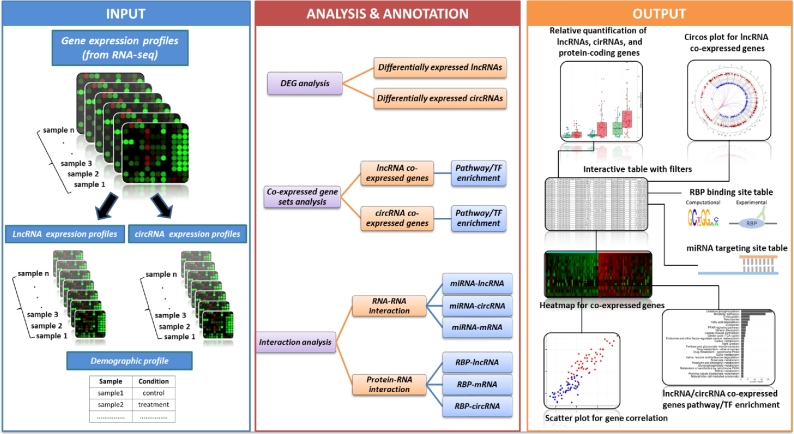
The overall design and the analytic workflow of circlncRNAnet.

Despite the enormous number of lncRNAs (∼15 000) annotated by GENCODE [23], our functional understanding of lncRNAs remains largely limited. While large-scale sequencing studies have become a standard approach for identifying candidate circRNAs/lncRNAs with significant expression alteration in certain cellular states, there may not be sufficient information in the literature to warrant further functional interrogation. Moreover, given the potentially widespread target spectrum of these ncRNAs as well as their extensive modes of action, a complete understanding of their biological relevance will depend on integrative analyses of systems data at various levels [24]. While a handful of publicly available databases have been reported (Table 1), they are quite limited in the scope of reference data and analytic modules, relying on existing datasets in public archives and annotating preselected regulatory features of ncRNAs. Thus, existing tools do not fully capture, from a network perspective, the functional implications of lncRNAs or circRNAs of interest. To solve this problem, we have implemented an integrative bioinformatics approach to examine *in silico* the functional networks of ncRNAs. The overall design and analytic workflow of this first “one-stop” web server tool for exploring the ncRNA biology are depicted in Fig. 1.

**Table 1:** Comparative functionalities of available web tools of ncRNAs.

Tool name	Interface	Both lncRNAs and circRNAs	Expression pattern	Co-expression: gene network	Co-expression: annotation/pathway	RBP binding site prediction	miRNA target prediction	Regulatory Network	Ref.
circlncRNAnet	Web server	Yes	Yes	Yes	Yes	Yes	Yes	Yes	This article
NONCODE	Web database		Yes						[53]
LNCipedia	Web database						Yes		[30]
ncFANs	Web server			Yes	Yes				[54]
lncRNAdb	Web database		Yes			Yes	Yes		[55]
LINC	R package			Yes	Yes				[56]
cogena	R package			Yes	Yes				[57]
WGCNA	R package			Yes					[37]
QUBIC	R package			Yes					[58]
circNet	Web database		Yes				Yes	Yes	[59]
CIRCpedia	Web database		Yes						[60]
Circ2Traits	Web database	Yes	Yes		Yes		Yes		[61]
CircInteractome	Web database					Yes	Yes	Yes	[62]
DeepBase V2.0	Web database	Yes	Yes						[24]
starBase V2.0	Web database	Yes			Yes	Yes	Yes	Yes	[63]

## Results and Methods

### Data input

**Figure 2:**
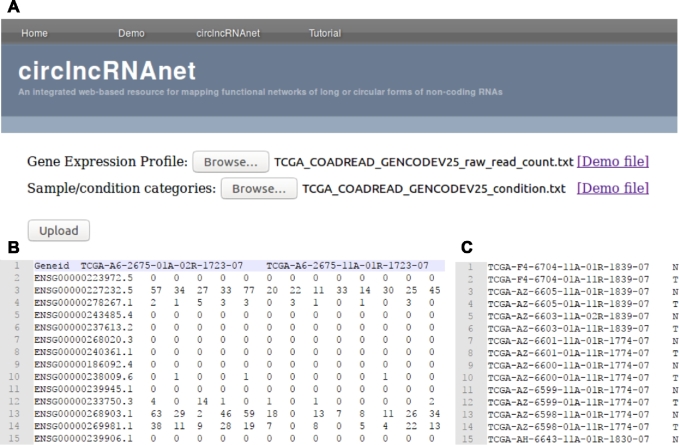
Input file formats for circlncRNAnet. Interface on the web server for data upload (A). Two files are uploaded prior to data analysis: a gene matrix table (B), which is generated using featureCounts, and a condition file describing the sample status (C).

To start, there are 2 separate upload pages for “lncRNA” and “circRNA” to meet the distinct analytic requirements of these 2 types of molecules (Fig. 2A). Users may upload tab-delimited text files that contain (1) expression matrix data of RNA-seq raw read counts, which are generated by using featureCounts (Fig. 2B) [25] and (2) sample/condition categories (Fig. 2C) into “Gene Expression Profile” and “Demographic Information,” respectively, on the webpage. For circRNA analyses, circRNA read counts, as quantified by KNIFE [26], should be additionally provided in a separate file. Procedures for processing the datasets into the appropriate format are outlined in the tutorial page on the web server [27]. For demonstration of use, 2 test datasets derived from publicly available RNA-seq data are included in the web server: The Cancer Genome Atlas (TCGA) data on colon and rectal adenocarcinoma (COAD and READ; for lncRNA) and the Encyclopedia of DNA Elements (ENCODE) data on the esophagus and sigmoid colon (for circRNA) [28, 29].

### Output summary

After the successful submission of a job, processing statuses, file format conversion, co-expression analysis, interactome networking, and report generation are displayed using a dynamic progress indicator. Computational tools and databases employed in this study are listed in Tables 2 and 3, respectively, which also outline the parameters used to carry out the corresponding analyses. The output section of the tutorial page [27] shows the standard output of circlncRNAnet based on the demonstration datasets. The standard output is represented by dynamic tables and charts, including bar and box plots, scatter plot, circos plot, heatmap, and network plots. Also included in the table is annotation information of the coding and noncoding genes, such as genome location, distance from query lncRNA or circRNA, lncRNA ID (ENCODE), coding potential [30], circRNA ID according to circBase [31], and circRNA (or host gene) splicing structure.

**Table 2:** Analytic and visualization R packages incorporated in circlncRNAnet

Analytic software	Version	Description	Ref.
circlize	0.4.1	Circos plot	[64]
clusterProfiler	3.2.14	Gene enrichment analysis	[65]
DESeq2	1.14.1	Differential expression analysis	[32]
factoextra	1.0.4	Principle component analysis	[66]
ggplot2	2.2.1.9000	Data visualization	[67]
plotly	4.7.1	Interactive data visualization	[68]
visNetwork	2.0.1	Network visualization	[69]
WGCNA	1.51	Correlation calculation	[37]

**Table 3:** List of databases and analytic tools employed by circlncRNAnet

Database	Version	Description	Parameters	Ref.
cisBP-RNA and Ray, 2013 (*Homo sapiens*)	2013	RNA binding protein motifs for FIMO to discover potential RNA binding sites	Downloaded from MEME motif database	[70]
dbNSFP (*Homo sapiens*)	3.2	Gene annotation	NA	[71]
ENCODE ChIP-Seq (*Homo sapiens*)	Feb 2017	Experimental transcription factor and protein binding sites	Regions from -3000∼1000 bp of TSS were considered as the promoter; in-house scripts were then used to collect peaks with >2 score and annotate as binding sites	[29]
ENCODE eCLIP (*Homo sapiens*)	Mar 2017	Experimental RNA binding protein binding sites	In-house scripts were used to collect all the peaks corresponding to binding sites; binding score for each target gene was represented by the lowest peak score	[29]
FIMO	4.11.2	Computational RNA binding protein binding sites discovering	Default	[44]
GENCODE (*Homo sapiens*)	Release 25	lncRNA annotation	NA	[23]
LNCipedia (*Homo sapiens*)	4	High-confidence lncRNA annotation	NA	[30]
miRanda	3.3a	miRNA binding sites detection	-m 10 000 000 -p 0.05	[72]
MSigDB	v5.2	Computational transcription factor and protein binding sites	The transcription factor targets dataset was used for TF enrichment analysis	[73]
RNAhybrid	2.1.2	miRNA binding sites detection	-sc 140, with cutoff seed similarity ≥85% and wobble pair similarity ≥85%	[48]
TarPmiR	Mar 2016	miRNA binding sites detection	-p 0.1	[74]

### Analytic module #1: coding–noncoding co-expression network profiling

After the upload, the server will first execute the differential expression analysis by using the R package DESeq2 [32]. The interactive interface allows users to define the candidate gene list by fold changes and *P*-value. Moreover, to inspect the expression distance between samples, principal component analysis (PCA) was implemented in our analysis pipeline.

**Figure 3:**
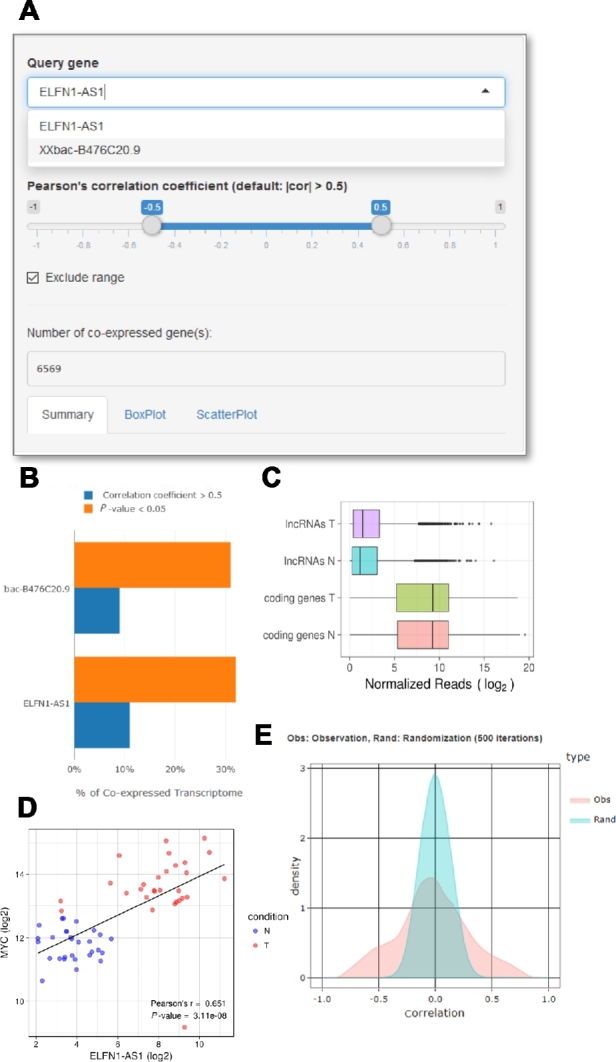
Schematic showing example outputs of circlncRNAnet analyses of lncRNA-based networks in colorectal cancer. After dataset upload, the server executes differential expression and expression correlation analyses. The web server allows the user to select query genes and correlation criteria (A). For an overview of the sequenced transcriptomes, the extent of the coordinated expression (B) and overall distribution of noncoding and coding RNA abundance (C) are displayed as summary graphs. As examples of use, co-expression network analysis of a known lncRNA, ELFN1-AS1, and a novel lncRNA, XXbac-B476C20.9, was performed using circlncRNAnet. (D) Scatter plot showing the extent of expression correlation between ELFNA-AS1 and 1 target, MYC. (E) Histogram displaying the distributions of the Pearson correlation coefficients of all ncRNA-mRNA pairs (Obs) and of a randomized correlation test (Rand).

**Figure 4:**
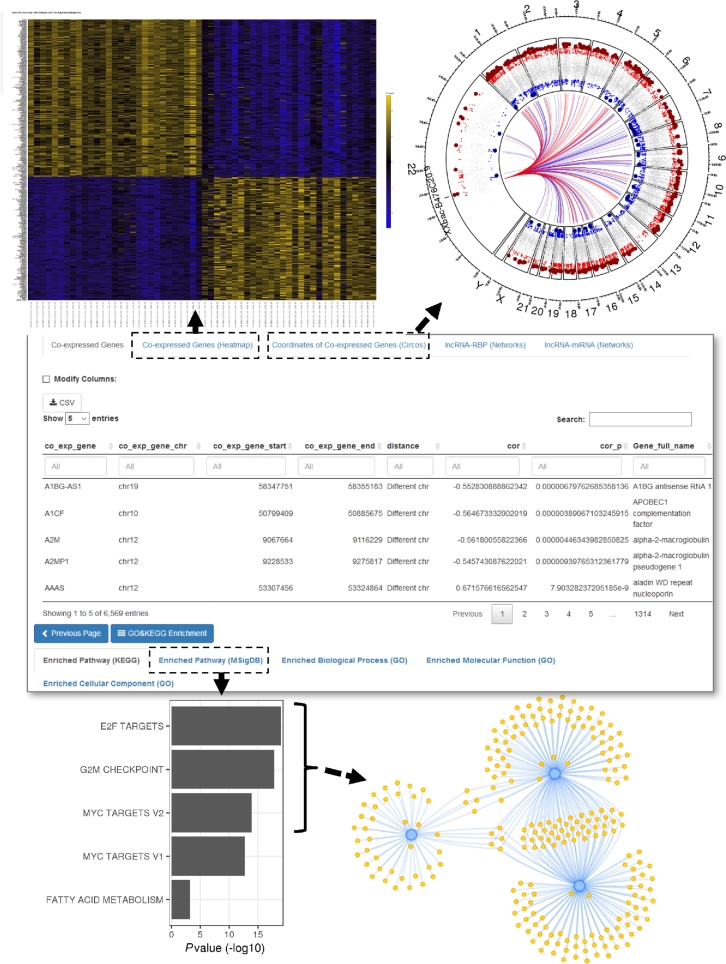
Additional examples of circlncRNAnet output of lncRNA-based networks in colorectal cancer. In addition to the analyses shown in Fig. 3, more options for network interrogation of ncRNA-based regulation can be accessed on the webpage (middle). For instance, heatmap representation of the genes co-expressed with ELFN1-AS1 (Pearson's |*r*| > 0.5) can be outputted (upper left). Pathway analysis of the co-expressed genes on the basis of MSigdb Hallmark pathways (bottom left), and its network depiction of the top 3 enriched pathways and the corresponding co-expressed components (bottom right). Circos plot can also be used to illustrate the genome-wide distribution of the top 100 co-expressed genes relative to the location of *XXbac-B476C20.9* (upper right).

Several known functional attributes of circRNAs/lncRNAs were taken into account when constructing this web server: First, we adopted the gene co-expression analysis, which is based on the concept of “guilt by association”—assuming that genes exhibiting analogous expression patterns may be involved in similar biological pathways, functions of unknown genes may be inferred *a priori* from the co-expressed, functionally known genes [33]. To this end, Wolfe et al. developed a method to demonstrate that co-expression with biologically defined modules may serve as a basis for characterizing the function of unknown genes [34]. Ricano-Ponce et al. also used co-expression analysis to deduce the function of lncRNAs with expression quantitative trait loci (eQTLs) effects [35]. The combined use of co-expression analysis and Gene Set Enrichment Analysis (GSEA) has been demonstrated to identify lncRNAs putatively involved in neuronal development [36]. To implement this co-expression analysis in circlncRNAnet, we used the R package WGCNA [37] to calculate the Pearson correlation coefficients of selected differentially expressed circRNA/lncRNA expression against all genes in the user-uploaded samples (Fig. 3A). For an overview of the sequenced transcriptomes, the extent of the coordinated expression (Fig. 3B) and overall distribution of noncoding and coding RNA abundance (Fig. 3C) can be displayed as summary graphs. To provide users with a guide in the selection of relevant criteria for expression correlation, the server displays a composite histogram showing the overall distribution of correlation coefficients calculated for all the ncRNA-mRNA pairs, superimposed with the results from randomized correlation tests (500 iterations of randomized Pearson correlations between target ncRNAs and 5000 randomly selected mRNAs). The highly correlated genes (based on user-defined Pearson's correlation) will also be subjected to pathway enrichment analysis (Fig. 4). The identity and enriched terms of the co-expression networks will be provided to facilitate further functional deduction of ncRNAs candidates.

As a proof of principle, we applied our analytic pipeline to a known example of cancer-associated lncRNAs, *ELFN1-AS1*. Kim et al. recently reported that MYC-regulated lncRNA *MYCLo-2* (also known as *ELFN1-AS1*) represses *CDKN2B* transcription coordinately with hnRNPK [38]. To demonstrate the utility of circlncRNAnet, we queried the functional network of *ELFN1-AS1*. We used TCGA data on COAD and READ and paired normal samples as the reference expression datasets. Co-expression gene network analysis for *ELFN1-AS1* may be done on the basis of the differentially expressed gene list and outputted according to user-defined criteria (Fig. 4, middle panel). To further visualize overall expression profiles of *ELFN1-AS1* co-expressed genes, “heatmap” may be used to display up to 500 of the most correlated genes (ranked by absolute *r* value) (Fig. 4, upper left panel). Pair-wise expression correlation between the ncRNA and co-expressed mRNA genes is also possible. For instance, as *ELFN1-AS1* is a known transcriptional target of MYC, users may compare the expression patterns between *ELFN1-AS1* and *MYC* in the TCGA data. This is done through “Scatter plot,” and enter “MYC” in the “Co-expressed gene” box (Fig. 3D). Next, for pathway analysis of genes co-expressed with *ELFN1-AS1*, the “GO & KEGG Enrichment” functionality is available, in which the “Enriched pathway (MSigDB)” will output top enriched pathways, together with a network representation of the components. In the case of *ELFN1-AS1*, MYC TARGETS V1 and MYC TARGETS V2 are shown as 2 of the top pathways, consistent with the previous findings (Fig. 4, lower panels).

In addition, we used another novel lncRNA as an example of our analytic approach. *XXbac-B476C20.9* was downregulated in colorectal cancer, and higher expression of *XXbac-B476C20.9* exhibited better survival expectancy, hinting at a tumor-suppressive role (data not shown). By using Pearson correlation analysis, we identified hundreds of genes that exhibit significant co-expression with this lncRNA (data not shown). By analyzing the chromosome distribution of *XXbac-B476C20.9* co-expressed genes, we did not see particular enrichment in chromosome 22 (where *XXbac-B476C20.9* locates) (Fig. 4, upper right panel), indicating that this lncRNA may not exert expression regulation in a cis manner.

Correlated expression may also be attributed to the functional interaction of the circRNAs/lncRNAs with particular transcription factor (TF) networks. Indeed, previous studies have reported that lncRNA could regulate TF activity through reciprocal interaction [39]. To address this possibility, our web server is equipped to determine whether the co-expression gene set is enriched in targets of specific TFs. Extensive TF-target pairs were first built by annotating 2 sources of data: (1) computational motif scan of TF binding sites and (2) experimental TF binding sites as archived by the ENCODE Chromatin immunoprecipitation sequencing (ChIP-Seq) data. For the latter, we retrieved ENCODE ChIP-seq data and defined the promoter region as a window from -3000 bp to +1000 bp of the transcription start site to establish putative TF occupancy. The output of this type of analysis can be accessed via gene enrichment module.

### Analytic module #2: RBP interactome mapping

Second, based on the lncRNAs that have been reported thus far, they have been mostly implicated in several aspects of gene expression, such as RNA stability, miRNA sponging, regulation of transcription factor, and epigenetic and chromosomal architecture [4, 7, 20, 21, 40]. Interestingly, behind these regulatory actions, molecular interactions are the most crucial determinant in lncRNAs’ roles. In this context, lncRNAs are known to associate with various proteins (i.e., RNA-binding proteins and chromatin modifiers). For example, lncRNA *ELFN1-AS1* interacts with hnRNPK to transcriptionally suppress the expression of *CDKN2B*, a tumor suppressor gene [38]. LncRNA *NORAD* acts as sequester of PUM2 to maintain genomic stability [41]. A colorectal cancer (CRC) associated lncRNA MYU binds hnRNPK and consequently stabilizes CDK6, which is critical for colon cancer cells’ growth [42]. These findings thus suggest that delineating the lncRNA-interacting protein network may effectively prompt the functional exploration of lncRNA candidates. In our efforts of mapping the protein interactome of lncRNAs, we have extensively curated and integrated 2 types of public data into reference annotations for the analytic workflow: computational RNA binding protein (RBP) motif scan and experimental RBP databases.

For this purpose, we first collected RBP binding motifs from MEME, which is a motif-discovering software, in addition to several RBP motifs from published data [43]. Next, we generated all lncRNA sequences from GENCODE, Release 25, and used FIMO to scan computationally for the presence of possible RBP binding sites [44]. For the empirical RBP sites, we retrieved the RBP binding sequences from ENCODE eCLIP [45]. To complement the repertoire of RBP included in the analysis, we also integrated protein interaction profile sequencing (PIP-seq) [46]. Although the footprints of protein binding do not readily reveal the identity of the associated factors, PIP-seq data may serve as evidence for molecular interaction.

**Figure 5:**
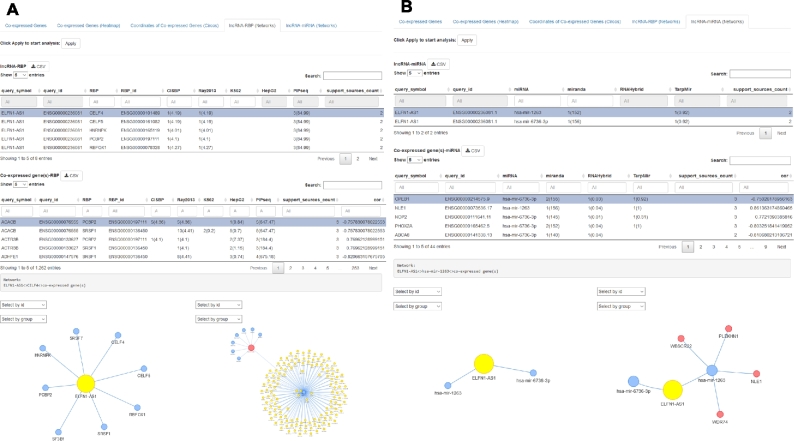
Examples of lncRNA-associated molecular components uncovered by circlncRNAnet. circlncRNAnet may be used to extensively profile the molecular interactome of candidate circRNAs/lncRNAs based on the compiled databases, with ELFN1-AS1 shown as an example in this figure. (A) For the RBP components, the interactome will be outputted in both the table format (top) and network configuration (bottom). (B) Similarly, for the putative miRNA sponge network, predicted ELFN1-AS1-targeting miRNAs are shown in table (top) and network (bottom) formats. The web server is also designed to construct the ncRNA-RBP-mRNAs or ncRNA-miRNA-mRNAs regulatory hierarchy. circlncRNAnet delineates co-expressed mRNA genes with mutually shared RBP binding or miRNA targeting sites. Consequently, an intersected gene list is compiled (top) and may be depicted in a 2-tier network configuration (bottom).

Given that our exemplary lncRNA *ELFN1-AS1* reportedly mediates its function through interacting with hnRNPK, we next tested whether this attribute could be recapitulated by circlncRNAnet. To interrogate the *ELFN1-AS1*-associated proteins, the “Retrieve lncRNA-binding protein” module can be selected to display a *ELFN1-AS1*-associated RNA-binding protein network (Fig. 5A). An RBP is considered a hit (i.e., potential interactor of the given lncRNA/circRNA) if its annotated motifs from at least 2 database sources are detected in the transcript sequence, and will be labeled with a gene symbol and a larger node size. The output of this demo analysis illustrates a number of putative interacting RBPs, one of which is HNRNPK, as reported (Fig. 5A).

### Analytic module #3: ceRNA networking

Third, aside from protein interactors, the role of circRNAs/lncRNAs in microRNA (miRNA)-mediated post-transcriptional regulation has emerged. By virtue of the distinct distribution of recurring miRNA target sequences in lncRNA transcripts, certain lncRNAs are known to compete with mRNA transcripts for complementary binding by the cognate miRNAs. This regulatory process, referred to as miRNA sponge or competing endogenous RNAs (ceRNAs) [47], alters the endogenous silencing activity of miRNAs, thereby impacting the expression of targeted mRNAs. Some lncRNAs have even been demonstrated as miRNA sponges in certain oncogenic processes [11, 12]. Thus, to complete this bioinformatics package, we installed in this web server an analytic module for sequence-based delineation of potential lncRNA-miRNA sponge pairs. Given that existing miRNA targeting sites databases annotate target sequences only in 3’ UTR, information regarding miRNA: ncRNA complementarity is not readily available. To resolve this issue, we generated a reference database that catalogs putative miRNA binding sites within lncRNAs/circRNAs as computationally predicted by 3 different miRNA target prediction tools (RNAhybrid, miRanda, and TarPmiR) [48–50]. Analogous to the RBP module, an miRNA target is considered a positive hit if 2 of the 3 software tools uncover its existence, and will be denoted as a larger node and shown with a gene symbol in the network diagram.

For the RNA components of the *ELFN1-AS1* interactomes, circlncRNAnet provides information on the putative miRNA targeting sites within the RNA sequences. To explore, the “miRNA targeting sites network” may be selected to show the corresponding network (Fig. 5B). Analogous to the RBP network, any miRNA target sequences predicted by at least 2 miRNA targeting site–discovering softwares (miRanda, RNAhybrid, and TarPmiR) will be labeled with gene symbols and a larger node size in the network (Fig. 5B).

### Analytic module #4: multitier regulatory hierarchy

mRNAs harboring the same miRNA binding sites as ncRNAs are likely to be subject to expression alteration in the miRNA sponge scenario—the inverse correlation in expression between miRNA and mRNAs/lncRNAs is expected [47]. Thus, to substantiate the putative miRNA sponge activity and also to delineate likely downstream mRNA targets, the web server is further designed to construct the ncRNA-miRNA-mRNAs regulatory hierarchy. For this purpose, 3’ UTRs with presumptive miRNA targeting, as revealed by the aforementioned prediction tools, will be cross-referenced with the gene set that shows correlated expression profiles with the candidate ncRNA. As a result, this intersected gene list presumably represents the targets of ncRNA-miRNA axis-mediated regulation, and will be depicted in a 2-tier network configuration (Fig. 5B).

Similar network analyses are available for decoding the ncRNA-RBP-mRNA network. To this end, a reference RBP-mRNA database was first established, in which all GENCODE mRNA genes were scanned and annotated for experimental and computational RBP binding using the above approaches. For a particular RBP in the ncRNA interactome that is selected by the user, all ncRNA-co-expressed mRNAs with mutual RBP binding will be assembled based on the RPB-mRNA database. These lines of information will then be integrated and subsequently outputted as the multitier molecular network (Fig. 5A).

### Benchmarking

circlncRNAnet is constructed on the Nginx 1.6.3 and Shiny 1.0.3 servers, which run on a CentOS 6.2 with 2 Intel XEON E5–2620 CPU and 200GB RAM. To optimize the CPU utilities for multiple users, we assign 2 threads for an analysis task. We tested the web service with 20 normal/tumor paired samples, for which the DESeq2 analysis required 130 seconds to produce differentially expressed genes. For calculating a co-expressed gene list, circlncRNAnet took 50 seconds for 1 query gene and 270 seconds for 10 query genes.

## Conclusions

With the expansion of transcriptome sequencing datasets, focusing on a select set of publicly available, but potentially irrelevant, sequencing data does not sufficiently address users’ research needs. This prompted us to build a completely new system with the flexibility of accepting private or public data. To further support efficient analyses and presentation, we have extensively curated public data into reference annotations for the circlncRNAnet workflow. Multilayer modules and algorithms then provide outputs on expression profiles, co-expression networks and pathways, and molecular interactomes, which are dynamically and interactively displayed according to user-defined criteria. In short, users may apply circlncRNAnet to obtain, in real time, multiple lines of functionally relevant information on the circRNAs/lncRNAs of their interest. The overall workflow takes only a few minutes, as compared with hours of manual effort of independent database searches and analyses. In summary, circlncRNAnet is the first of its kind in the regulatory RNA research field, providing a “one-stop” resource for in-depth analyses of ncRNA biology. A tutorial with demo datasets is available under “Tutorial,” in which the functional network of known lncRNA was illustrated *in silico* as an example.

## Availability of supporting source code and requirements

- Project name: circlncRNAnet
- Project home page: http://app.cgu.edu.tw/circlnc/[27], https://github.com/smw1414/circlncRNAnet [51]
- Operating system(s): platform independent
- Programming language: PHP, JavaScript, R, R shiny and Shell script
- Other requirements: JavaScript supporting web browser
- License: GPLv3
- Research Resource Identifier: circlncRNAnet, RRID:SCR_015794

## Availability of supporting data

The analytic modules and test datasets (from TCGA and ENCODE) are available in the GitHub repository [51]. An archival copy of the modules and test datasets is also available via the *GigaScience* repository, *Giga*DB [52]. For the convenience of prospective users, we also provided on GitHub instructions on running our pipeline in local mode.

## Abbreviations

ceRNA: competing endogenous RNA; ChIP-Seq: chromatin immunoprecipitation sequencing; circRNA: circular RNA; COAD: colon adenocarcinoma; CRC: colorectal cancer; GSEA: Gene Set Enrichment Analysis; lncRNAs: long noncoding RNA; miRNA: microRNA; mRNAs: messenger RNA; ncRNA: noncoding RNA; PIP-seq: Protein Interaction Profile sequencing; RBP: RNA-binding protein; READ: rectal adenocarcinoma; TCGA: The Cancer Genome Atlas.

## Funding

This work was supported by grants from the Ministry of Science and Technology of Taiwan (MOST104–2321-B-182–007-MY3 to P.J.H.; MOST106–2320-B-182–035-MY3 to H.L.; MOST104–2320-B-182–029-MY3 and MOST105–2314-B-182–061-MY4 to B.C.M.T.; MOST103–2632-B-182–001, MOST104–2632-B-182–001, and MOST105–2632-B-182–001), Chang Gung Memorial Hospital (CMRPD1G0321 and CMRPD1G0322 to P.J.H.; CMRPD1F0571 to H.L.; CMRPG3D1513 and CMRPG3D1514 to W.S.T.; CMRPD3E0153, CMRPD1F0442, and BMRP960 to B.C.M.T.), the National Health Research Institute of Taiwan (NHRI-EX105–10321SI), the Ministry of Education of Taiwan, and Biosignature Research Grant CIRPD3B0013 for supporting bioinformatics and computing resources.

## Competing interests

The authors declare that they have no competing interests.

## Author contributions

H.L. and B.C.T. conceived the original idea of the web server. S.W., P.H., Y.C., C.L., W.T., and H.L. designed and implemented the web server. S.W., P.J., and Y.C. conducted the benchmarks. C.L., C.Y., W.T., and B.C.T. tested the system and provided feedback on features and functionality. S.W., H.L., and B.C.T. wrote the manuscript. All authors read and approved the final manuscript.

## Supplementary Material

GIGA-D-17-00121_Original-Submission.pdf

GIGA-D-17-00121_Revision-1.pdf

Response-to-Reviewer-Comments_Original-Submission.pdf

Reviewer-1-Report-(Original-Submission) -- Tyler Weirick 20 Jun 2017 Reviewed

Reviewer-2-Report-(Original-Submission) -- Wilfried Haerty 01 Aug 2017 Reviewed

Reviewer-2-Report-(Revision-1) -- Wilfried Haerty 16 Oct 2017 Reviewed
