# Supplementary material for: circlncRNAnet: an integrated web-based resource for mapping functional networks of long or circular forms of noncoding RNAs
Source: Gigascience. 2017 Nov 29;7(1):gix118. doi: 10.1093/gigascience/gix118 (PMC5765557; doi:10.1093/gigascience/gix118)
Supplement: GIGA-D-17-00121_Original-Submission.pdf [file gix118_giga-d-17-00121_original-submission.pdf]

GigaScience

circlncRNAnet: An integrated web-based resource for mapping functional networks of long or circular forms of non-coding RNAs

--Manuscript Draft--

**Manuscript Number:**	GIGA-D-17-00121
**Full Title:**	circlncRNAnet: An integrated web-based resource for mapping functional networks of long or circular forms of non-coding RNAs
**Article Type:**	Technical Note
**Funding Information:**	Ministry of Science and Technology, Taiwan (TW) — Dr. Po-Jung Huang<br>Dr. Hsuan Liu<br>Dr. Bertrand Chin-Ming Tan
	Chang Gung Memorial Hospital, Linkou — Dr. Wen-Sy Tsai<br>Dr. Hsuan Liu<br>Dr. Bertrand Chin-Ming Tan
**Abstract:**	Despite their lack of protein-coding potential, lncRNAs and circRNAs have emerged as key determinant in gene regulation, acting to fine-tune transcriptional and signaling output. At the mechanistic level, regardless of the forms, these non-coding RNA transcripts have been known to impact expression of messenger RNAs (mRNAs) via epigenetic and post-transcriptional regulation. Given their widespread target spectrum as well as extensive modes of action, a complete understanding of their biological relevance will depend on integrative analyses of systems data at various levels. While a handful of publicly available databases have been reported, existing tools do not fully capture from a network perspective the functional implications of lncRNAs or circRNAs of interest. Through an integrated and streamlined design, circlncRNAnet is aimed to broaden the understanding of ncRNA candidates by testing in silico several hypotheses of ncRNA-based functions on the basis of large-scale RNA-seq data. It is implemented with several features representing advances in the bioinformatics of ncRNAs: 1) a flexible framework that accepts and processes user-defined NGS-based expression data; 2) multiple analytic modules that assigns and productively assesses the regulatory networks of user-selected ncRNAs by cross-referencing extensively curated databases; 3) an all-purpose, information-rich workflow design that is tailored to all types of ncRNAs. Outputs on expression profiles, co-expression networks & pathways, and molecular interactomes, are dynamically and interactively displayed according to user-defined criteria. In short, users may apply circlncRNAnet to obtain, in real time, multiple lines of functionally relevant information on the circRNAs/lncRNAs of their interest. In summary, circlncRNAnet is the first of its kind in the regulatory RNA research field, providing a "one-stop" resource for in-depth analyses of ncRNA biology.
**Corresponding Author:**	Bertrand Tan, Ph.D.<br>Chang Gung University<br>Tao-Yuan, TAIWAN
**Corresponding Author Secondary Information:**	
**Corresponding Author's Institution:**	Chang Gung University
**Corresponding Author's Secondary Institution:**	
**First Author:**	Shaomin Wu
**First Author Secondary Information:**	
**Order of Authors:**	Shaomin Wu
	Po-Jung Huang
	Yi-Feng Chang
	Chi-Ching Lee

	Chia-Yu Yang
	Wen-Sy Tsai
	Hsuan Liu
	Bertrand Chin-Ming Tan, Ph.D.
**Order of Authors Secondary Information:**	
**Opposed Reviewers:**	
**Additional Information:**	
**Question**	**Response**
Are you submitting this manuscript to a special series or article collection?	No
**Experimental design and statistics**<br><br>Full details of the experimental design and statistical methods used should be given in the Methods section, as detailed in our Minimum Standards Reporting Checklist. Information essential to interpreting the data presented should be made available in the figure legends.<br><br>Have you included all the information requested in your manuscript?	Yes
**Resources**<br><br>A description of all resources used, including antibodies, cell lines, animals and software tools, with enough information to allow them to be uniquely identified, should be included in the Methods section. Authors are strongly encouraged to cite Research Resource Identifiers (RRIDs) for antibodies, model organisms and tools, where possible.<br><br>Have you included the information requested as detailed in our Minimum Standards Reporting Checklist?	Yes
**Availability of data and materials**<br><br>All datasets and code on which the conclusions of the paper rely must be either included in your submission or deposited in publicly available repositories (where available and ethically appropriate), referencing such data using a unique identifier in the references and in the "Availability of Data and Materials" section of your manuscript.	Yes

Have you have met the above requirement as detailed in our Minimum Standards Reporting Checklist?	

Manuscript

Click here to download Manuscript circlncRNAnet draft_wu_20170530.docx

circlncRNAnet: An integrated web-based resource for mapping functional networks of long or circular forms of non-coding RNAs

Shao-Min Wu[1,*], Po-Jung Huang[2,3,4,*], Yi-Feng Chang[3,*], Chi-Ching Lee[5], Chia-Yu Yang[1,3,6,7], Wen-Sy Tsai[7], Hsuan Liu[1,3,7,8], Bertrand Chin-Ming Tan[1,2,9]
[1]Graduate Institute of Biomedical Sciences, College of Medicine, Chang Gung University, Kueishan, Taoyuan, Taiwan; [2]Department of Biomedical Sciences, College of Medicine, Chang Gung University, Kueishan, Taoyuan, Taiwan; [3]Molecular Medicine Research Center, Chang Gung University, Kueishan, Taoyuan, Taiwan; [4]Genomic Medicine Core Laboratory, Chang Gung Memorial Hospital, Linkou, Taiwan; [5]Department of Computer Science and Information Engineering, College of Engineering, Chang Gung University, Kueishan, Taoyuan, Taiwan; [6]Department of Microbiology and Immunology, College of Medicine, Chang Gung University, Kueishan, Taoyuan, Taiwan; [7]Division of Colon and Rectal Surgery, Department of Surgery, Chang Gung Memorial Hospital, Linkou, Taiwan; [8]Department of Cell and Molecular Biology, College of Medicine, Chang Gung University, Kueishan, Taoyuan, Taiwan; [9]Department of Neurosurgery, Lin-Kou Medical Center, Chang Gung Memorial Hospital, Linkou, Taiwan

*These authors contributed equally to this work.
Correspondence to:
Bertrand Chin-Ming Tan, Graduate Institute of Biomedical Sciences and Department of Biomedical Sciences, College of Medicine, Chang Gung University, 259 Wen-Hwa First Road, Kwei-San, Tao-Yuan 333, Taiwan, ROC. Tel.: +886-3-211-8800; Fax: +866-3-211-8700; E-mail: btan@mail.cgu.edu.tw
Hsuan Liu, E-mail: liu-hsuan@mail.cgu.edu.tw
Wen-Sy Tsai, E-mail: wensy@adm.cgmh.org.tw

Abstract

Background

Despite their lack of protein-coding potential, lncRNAs and circRNAs have emerged as key determinant in gene regulation, acting to fine-tune transcriptional and signaling output. At the mechanistic level, regardless of the forms, these non-coding RNA transcripts have been known to impact expression of messenger RNAs (mRNAs) via epigenetic and post-transcriptional regulation. Given their widespread target spectrum as well as extensive modes of action, a complete understanding of their biological relevance will depend on integrative analyses of systems data at various levels.

Findings

While a handful of publicly available databases have been reported, existing tools do not fully capture from a network perspective the functional implications of lncRNAs or circRNAs of interest. Through an integrated and streamlined design, circlncRNAnet is aimed to broaden the understanding of ncRNA candidates by testing *in silico* several hypotheses of ncRNA-based functions on the basis of large-scale RNA-seq data. It is implemented with several features representing advances in the bioinformatics of ncRNAs: 1) a flexible framework that accepts and processes user-defined NGS-based expression data; 2) multiple analytic modules that assigns and productively assesses the regulatory networks of user-selected ncRNAs by cross-referencing extensively curated databases; 3) an all-purpose, information-rich workflow design that is tailored to all types of ncRNAs. Outputs on expression profiles, co-expression networks & pathways, and molecular interactomes, are dynamically and interactively displayed according to user-defined criteria.

Conclusions

In short, users may apply circlncRNAnet to obtain, in real time, multiple lines of functionally relevant information on the circRNAs/lncRNAs of their interest. In summary, circlncRNAnet is the first of its kind in the regulatory RNA research field, providing a "one-stop" resource for in-depth analyses of ncRNA biology. circlncRNAnet is freely available at http://app.cgu.edu.tw/circlnc/.

Keywords

lncRNAs – circRNAs – co-expression network – molecular interactome

Introduction

In contrast to the 1% of human genome that encodes proteins, 70% to 90% of the genome can actually be transcribed at some point during development – this generates a large transcriptome of non-coding RNAs (ncRNA), part of which ultimately yield definite short or long RNAs with limited protein-coding capacity (1). In recent years, deep sequencing technologies have unraveled the non-coding constituents of the transcriptome, most notably lncRNAs and circRNAs. Despite the lack of protein-coding potential, these once uncharted parts have emerged as a key determinant in gene regulation, acting as critical switches that fine-tune transcriptional and signaling output (2,3).

Distinct from small non-coding RNAs such as microRNAs and snRNAs, long non-coding RNAs (lncRNAs) are RNA molecules with length of longer than 200 nucleotides but lack detectable open reading frame (4). They are usually transcribed by RNA polymerase II and exhibit known attributes of the messenger RNAs, such as post-transcriptional processing. Circular RNAs (circRNAs) are a more recently discovered class of non-coding RNAs, which is defined not by length but rather the unique structure of covalently closed circularity (5,6). Despite their differences in structure and biosynthesis steps, lncRNAs and circRNAs are much more common in terms of their roles and mechanisms in gene regulation. Even in the absence of the protein products, these RNA molecules have been found to associate with distinct cellular compartments or components, and may act *in cis* or *trans* in target gene regulation, (7-10): At the epigenetic and transcriptional level, lncRNAs are known to interact with transcriptional activator or repressor and consequently impact transcriptional efficiency. By binding with the chromatin-modifying factors, lncRNAs could also serve as guide or scaffold that controls the epigenetic status. At the post-transcription level, lncRNAs may bind to target RNAs and alter transcript structure, splicing pattern and stability. Both lncRNAs and circRNAs have been found to harbor microRNA response elements (MREs) and potentially act as “miRNA sponges” that sequester these endogenous small RNAs. They are therefore part of the competing endogenous RNA (ceRNA) network with the potential to alter the miRNA-targeted mRNA expression (8,11,12). Another mode of regulation exerted by lncRNAs is their associations with RNA-binding proteins. Similar to the ceRNA scenario, this molecular interaction may impact the localization, and thus activity, of these gene regulators. Finally, in line with their critical roles as gene regulators, both circRNAs and lncRNAs exhibit unique expression profiles in various human cancers, suggestive of a correlation with disease progression and possibly its value as predictor of patient outcome (13-17). Delineation of these transcriptomic networks therefore is of importance in understanding ncRNAs and associated biological processes and may shed new light on diseases and possibly new avenues of therapeutic interventions (18-20).

Despite the enormous number of lncRNAs (~15,000) annotated by the GENCODE (http://www.gencodegenes.org/), functional understanding of the lncRNAs remains largely limited. While large-scale sequencing studies have become a standard approach for identifying candidate circRNAs/lncRNAs with significant expression alteration in certain cellular states, there may not be sufficient information in the literature to warrant further functional interrogation. Moreover, given the potentially widespread target spectrum of these ncRNAs as well as their extensive modes of action, a complete understanding of their biological relevance will depend on

integrative analyses of systems data at various levels (21). Towards this end, while a handful of publicly available databases have been reported (Table 1), they are quite limited in the scope of reference data and analytic modules, relying on existing datasets in public archives and annotating pre-selected regulatory features of ncRNAs. Thus, existing tools do not fully capture from a network perspective the functional implications of lncRNAs or circRNAs of interest. To circumvent this problem, we have implemented an integrative bioinformatics approach to examine *in silico* the cellular roles of lncRNAs by mapping their functional networks. The overall design and the analytic workflow of this first "one-stop" web server tool for exploring the ncRNA biology are depicted in Figure 1.

Results and Methods

Data Input

To start, there are two separate upload pages for "lncRNA" and "circRNA" to meet distinct analytic requirements of these two types of molecules (Fig. 2A). Users may upload tab-delimited text files that contain 1) expression matrix data of RNA-seq raw read counts, which are generated by using featureCounts (Fig. 2B) and 2) sample/condition categories (Fig. 2C), respectively into "Gene Expression Profile" and "Demographic Information" on the webpage. For circRNA analyses, circRNA read counts, as quantified by KNIFE, should be additionally provided in a separate file. Procedures for processing the datasets into the appropriate format are outlined in the tutorial page on the web server (http://app.cgu.edu.tw/circlnc/). For demonstration of use, two test datasets derived from publicly available RNA-seq data are included in the webserver: Cancer Genome Atlas (TCGA) data on colon and rectal adenocarcinoma (COAD and READ) (for lncRNA) and ENCODE's data on esophagus and sigmoid colon (for circRNA).

Output summary

After the successful submission of a job, processing statuses, file format conversion, co-expression analysis, interactome networking, and report generation, are displayed using a dynamic progress indicator. The output section of tutorial page (http://app.cgu.edu.tw/circlnc/) shows the standard output of circlncRNAnet based on the demonstration datasets. The standard output is represented by dynamic tables and charts, including bar and box plots, scatter plot, circos plot, heatmap, and network plots. Also included in the table is annotation information of the coding and non-coding genes, such as genome location, distance from query lncRNA or circRNA, lncRNA ID (ENCODE), coding potential (22), circRNA ID according to circBase (23), circRNA (or host gene) splicing structure.

Analytic module #1: coding–non-coding co-expression (CNC) network profiling

After the upload, the server will first execute the differential expression analysis. The interactive interface allows users to define the candidate gene list by fold changes and p-value. Moreover, to inspect the expression distance between samples, principal components analysis (PCA) was implemented in our analysis pipeline.

Several known functional attributes of circRNAs/lncRNAs were taken into account when constructing this web server: First, we adopted the gene co-expression analysis, which is based on the concept of "guilt by association" – assuming that genes exhibiting analogous expression patterns may be involved in similar biological pathways, functions of unknown genes may be inferred *a priori* from the co-

expressed, functionally known genes (24). To this end, Wolfe et al. developed a method to demonstrate that co-expression with biologically defined modules may serve as a basis for characterizing the function of unknown genes (25). Ricano-Ponce et al. also used co-expression analysis to deduce the function of lncRNAs with expression quantitative trait loci (eQTLs) effects (26). Combined use of co-expression analysis and Gene Set Enrichment Analysis (GSEA) has been demonstrated to identify lncRNAs putatively involved in neuronal development (27). To implement this analysis in circlncRNAnet, we will calculate Pearson correlation of selected circRNAs/lncRNAs expression against all sequenced genes in the user-uploaded samples (Fig. 3A). For an overview of the sequenced transcriptomes, extent of the coordinated expression (Fig. 3B) and overall distribution of non-coding and coding RNA abundance (Fig. 3C) can be displayed as summary graphs. The highly correlated genes (based on user-defined Pearson's correlation) will also be subjected to pathway enrichment analysis (Fig. 4). The identity and enriched terms of the co-expression networks will be provided to facilitate further functional deduction of ncRNAs candidates.

As a proof of principle, we applied our analytic pipeline to a known example of cancer-associated lncRNAs, *ELFN1-AS1*. Kim *et al.* recently reported that MYC-regulated lncRNA *MYCLo-2* (also known as *ELFN1-AS1*) represses *CDKN2B* transcription coordinately with hnRNPK (28). To demonstrate the utility of circlncRNAnet, we queried the functional network of *ELFN1-AS1*. We used The Cancer Genome Atlas (TCGA) data on COAD and READ and paired normal samples as the reference expression datasets. Co-expression gene network analysis for *ELFN1-AS1* may be done on the basis of the differentially expressed gene list and outputted according to user-defined criteria (Fig. 4, middle panel). To further visualize overall expression profiles of *ELFN1-AS1* co-expressed genes, "heatmap" may be used to display up to 500 of most correlated genes (ranked by absolute r value) (Fig. 4, upper left panel). Pair-wise expression correlation between the ncRNA and co-expressed mRNA genes is also possible. For instance, as *ELFN1-AS1* is a known transcriptional target of MYC, users may compare the expression patterns between *ELFN1-AS1* and *MYC* in the TCGA data. This is done through "Scatter plot" and enter "MYC" in the "Co-expressed gene" box (Fig. 3D). Next, pathway analysis of genes co-expressed with *ELFN1-AS1*, the "GO & KEGG Enrichment" functionality is available, in which the "Enriched pathway (MSigDB)" will output top enriched pathways, together with a network representation of the components. In the case of *ELFN1-AS1*, MYC TARGETS V1 and MYC TARGETS V2 are shown as two of the top pathways, consistently with the previous findings (Fig. 4, lower panels).

In addition, we used another novel lncRNA as an example of our analytic approach. *XXbac-B476C20.9* was downregulated in the colorectal cancer, and higher expression of *XXbac-B476C20.9* exhibited better survival expectancy, hinting at a tumor-suppressive role (data not shown). By using Pearson correlation analysis, we identified hundreds of genes that exhibit significant co-expression with this lncRNA (data not shown). By analyzing the chromosome distribution of *XXbac-B476C20.9* co-expressed genes, we did not see particular enrichment in chromosome 22 (where *XXbac-B476C20.9* locates) (Fig. 4, upper right panel), indicating that this lncRNA may not exert expression regulation in a cis manner.

Correlated expression may also be attributed to the functional interaction of the

circRNAs/lncRNAs with particular transcription factor (TF) networks. Indeed, previous studies have reported that lncRNA could regulate TF activity through reciprocal interaction (29). To address this possibility, our web server is equipped to determine whether the co-expression gene set is enriched in targets of specific TFs. Extensive TF-target pairs were first built by annotating two sources of data: 1) computational motif scan of TF binding sites, and 2) experimental TF binding sites as archived by the ENCODE ChIP data. For the latter, we retrieved ENCODE ChIP-seq data and defined the promoter region as a window from -3000 bp to +1000 bp of the transcription start site to establish putative TF occupancy. Output of this type of analysis can be accessed via gene enrichment module.

Analytic module #2: RBP interactome mapping

Second, based on the lncRNAs that have been reported thus far, they have been mostly implicated in several aspects of gene expression, such as RNA stability, miRNA sponging, regulation of transcription factor, epigenetic and chromosome architecture (4,7,18,19,30). Interestingly, behind these regulatory actions, molecular interactions are the most crucial determinant in lncRNAs' roles. In this context, lncRNAs are known to associate with various proteins (i.e. RNA-binding proteins and chromatin modifiers). For example, lncRNA *ELFN1-AS1* interacts with hnRNPK to transcriptionally suppress the expression of *CDKN2B,* a tumor suppressor gene (28). LncRNA *NORAD* acts as sequester of PUM2 to maintain genomic stability (31). A CRC-associated lncRNA MYU binds hnRNPK and consequently stabilizes CDK6, which is critical for colon cancer cells growth (32). These findings thus suggest that delineating the lncRNA-interacting protein network may effectively prompt the functional exploration of lncRNA candidates. In our efforts of mapping the protein interactome of lncRNAs, we have extensively curated and integrated two types of public data into reference annotations for the analytic workflow: computational RNA binding protein (RBP) motif scan and experimental RBP databases.

For this purpose, we first collected RBP binding motifs from MEME, which is a motif discovering software, in addition to several RBP motifs from published data (33). Next, we generated all lncRNAs sequences from GENCODE v25 and used FIMO to scan computationally for the presence of possible RBP binding sites (34). For the empirical RBP sites, we retrieved the RBP binding sequences from ENCODE eCLIP (35). To complement the repertoire of RBP included in the analysis, we also integrated protein interaction profile sequencing (PIP-seq) (36). Although the footprints of protein binding do not readily reveal the identity of the associated factors, PIP-seq data may serve as evidence for molecular interaction.

Given that our exemplary lncRNA *ELFN1-AS1* reportedly mediates its function through interacting with hnRNPK, we next tested whether this attribute could be recapitulated by circlncRNAnet. To interrogate the *ELFN1-AS1*-associated proteins, the "Retrieve lncRNA-binding protein" module can be selected to display *ELFN1-AS1*-associated RNA-binding protein network (Fig. 5A). A RBP is considered a hit (i.e. potential interactor of the given lncRNA/circRNA) if its annotated motifs from at least two database sources are detected in the transcript sequence, and will be labeled with gene symbol and a larger node size. The output of this demo analysis illustrates a number of putative interacting RBPs, one of which is HNRNPK, as reported (Fig. 5A).

Analytic module #3: ceRNA networking

Third, aside from protein interactors, the role of circRNAs/lncRNAs in microRNA (miRNA)-mediated post-transcriptional regulation has emerged. By virtue of the distinct distribution of recurring miRNA target sequences in lncRNA transcripts, certain lncRNAs are known to compete with mRNA transcripts for complementary binding by the cognate miRNAs. This regulatory process, referred to as miRNA sponge or competing endogenous RNAs (ceRNAs) (37), alters the endogenous silencing activity of miRNAs, thereby impacting the expression of targeted mRNAs. Some lncRNAs have even been demonstrated as miRNA sponge in certain oncogenic processes (11,12). Thus, to complete this bioinformatics package, we installed in this web server an analytic module for sequence-based delineation of potential lncRNA-miRNA sponge pairs. Given that existing miRNA targeting sites databases annotate target sequences only in 3' UTR, information regarding miRNA:ncRNA complementarity is not readily available. To resolve this issue, we generated a reference database that catalogues putative miRNA binding sites within lncRNAs/circRNAs as computationally predicted by three different miRNA target prediction tools (RNAhybrid, miRanda and TarPmiR) (38-40). Analogous to the RBP module, a miRNA target is considered a positive hit if two of the three software tools uncover its existence, and will be denoted as larger node and shown with gene symbol in the network diagram.

For the RNA components of the *ELFN1-AS1* interactomes, circlncRNAnet provides information on the putative miRNA targeting sites within the RNA sequences. To explore, the "miRNA targeting sites network" may be selected to show the corresponding network (Fig. 5B). Analogous to the RBP network, any miRNA target sequences predicted by at least two miRNA targeting site discovering software (miRanda, RNAhybrid and TarPmiR) will be labeled with gene symbols and larger node size in the network (Fig. 5B).

Analytic module #4: Multi-tier regulatory hierarchy

mRNAs harboring the same miRNA binding sites as ncRNAs are likely to be subject to expression alteration in the miRNA sponge scenario – the inverse correlation in expression between miRNA and mRNAs/lncRNAs is expected (37). Thus, to substantiate the putative miRNA sponge activity and also to delineate likely downstream mRNA targets, the web server is further designed to construct the ncRNA-miRNA-mRNAs regulatory hierarchy. For this purpose, 3' UTRs with presumptive miRNA targeting as revealed by the aforementioned prediction tools will be cross-referenced with the gene set that show correlated expression profiles with the candidate ncRNA. As a result, this intersected gene list presumably represent the targets of ncRNA-miRNA axis-mediated regulation, and will be depicted in a two-tier network configuration (Fig. 5B).

Similar network analyses are available for decoding the ncRNA-RBP-mRNA network. To this end, a reference RBP-mRNA database was first established, in which all GENCODE mRNA genes were scanned and annotated for experimental and computational RBP binding using the above approaches. For a particular RBP in the ncRNA interactome that is selected by the user, all ncRNA-co-expressed mRNAs with mutual RBP binding will be assembled based on the RPB-mRNA database. These lines of information will then be integrated and subsequently outputted as the multi-tier molecular network (Fig. 5A).

Benchmarking

circlncRNAnet is constructed on the Nginx 1.6.3 and Shiny 1.0.3 server, which runs on a CentOS 6.2 with two Intel XEON E5-2620 CPU and 200GB RAM. To optimize the CPU utilities for multiple users, we assign two threads for an analysis task. We tested the web service with 20 normal/tumor paired samples, for which the DESeq2 analysis required 130 seconds to produce differentially expressed genes. For calculating co-expressed gene list, circlncRNAnet took 50 seconds for one query gene and 270 seconds for ten query genes.

Conclusions

With the expansion of transcriptome sequencing datasets, focusing on a select set of publicly available, but potentially irrelevant, sequencing data does not sufficiently address users' research needs. This prompted us to build a completely new system with the flexibility of accepting private or public data. To further support efficient analyses and presentation, we have extensively curated public data into reference annotations for the circlncRNAnet workflow. Multi-layer modules and algorithms then provide outputs on expression profiles, co-expression networks & pathways, and molecular interactomes, which are dynamically and interactively displayed according to user-defined criteria. In short, users may apply circlncRNAnet to obtain, in real time, multiple lines of functionally relevant information on the circRNAs/lncRNAs of their interest. The overall workflow takes only a few minutes, as compared to hours of manual efforts of independent database searches and analyses. In summary, circlncRNAnet is the first of its kind in the regulatory RNA research field, providing a "one-stop" resource for in-depth analyses of ncRNA biology. A tutorial with demo datasets is available under "Tutorial", in which the functional network of known lncRNA was illustrated *in silico* as an example.

Availability of supporting source code and requirements

- Project name: circlncRNAnet
- Project home page: http://app.cgu.edu.tw/circlnc/
  https://github.com/smw1414/circlncRNAnet
- Operating system(s): Platform independent
- Programming language: PHP, JavaScript, R, R shiny and Shell script
- Other requirements: JavaScript supporting web browser
- License: GPLv3

Availability of supporting data

The analytic modules and test datasets (from TCGA and ENCODE) are available in the GitHub repository (41) and *GigaScience* GigaDB repository.

Declarations

Abbreviations

ceRNA: competing endogenous RNA; circRNA: circular RNA; CNC: coding–non-coding co-expression; COAD: Colon adenocarcinoma; GSEA: Gene Set Enrichment Analysis; lncRNAs: long non-coding RNA; miRNA: microRNA; mRNAs: messenger RNA; ncRNA: non-coding RNA; PIP-seq: Protein Interaction Profile sequencing; RBP: RNA-binding protein; READ: Rectal adenocarcinoma; TCGA: The Cancer Genome Atlas.

Acknowledgements

We are grateful to members of the BC-MT laboratory for critical reading of the article and important discussions. This work was supported by grants from the Ministry of Science and Technology of Taiwan (MOST104-2321-B-182-007-MY3 to P-JH; MOST105-2320-B-182-017 to HL; MOST104-2320-B-182-029-MY3 and MOST105-2314-B-182-061-MY4 to BC-MT; MOST103-2632-B-182-001, MOST104-2632-B-182-001, and MOST105-2632-B-182-001), Chang Gung Memorial Hospital (CMRPD1F0571 to HL; CMRPG3D1511, CMRPG3D1512, and CMRPG3D1513 to W-ST; CMRPD1C0843, CMRPD3E0153, CMRPD1F0441, and BMRP960 to BC-MT), the National Health Research Institute of Taiwan (NHRI-EX105-10321SI), the Ministry of Education of Taiwan, and Biosignature Research Grant CIRPD3B0013 for supporting bioinformatics and computing resources.

Authors' contributions

HL and BCT conceived the original idea of the webserver. SW, PH, YC, CL, WT and HL designed and implemented the webserver. SW, PJ and YC conducted the benchmarks. CL, CY, WT and BCT tested the system, provided feedback on features and functionality. SW, HL and BCT wrote the manuscript. All authors read and approved the final manuscript.

Competing interests

The authors declare that they have no competing interests

References

1. Ponting, C.P., Oliver, P.L. and Reik, W. (2009) Evolution and functions of long noncoding RNAs. *Cell*, **136**, 629-641.
2. Liu, J., Liu, T., Wang, X. and He, A. (2017) Circles reshaping the RNA world: from waste to treasure. *Mol Cancer*, **16**, 58.
3. Quinn, J.J. and Chang, H.Y. (2016) Unique features of long non-coding RNA biogenesis and function. *Nat Rev Genet*, **17**, 47-62.
4. Wang, K.C. and Chang, H.Y. (2011) Molecular mechanisms of long noncoding RNAs. *Mol Cell*, **43**, 904-914.
5. Jeck, W.R. and Sharpless, N.E. (2014) Detecting and characterizing circular RNAs. *Nat Biotechnol*, **32**, 453-461.
6. Petkovic, S. and Muller, S. (2015) RNA circularization strategies in vivo and in vitro. *Nucleic Acids Res*, **43**, 2454-2465.
7. Rinn, J.L. and Chang, H.Y. (2012) Genome regulation by long noncoding RNAs. *Annu Rev Biochem*, **81**, 145-166.
8. Wang, X., Arai, S., Song, X., Reichart, D., Du, K., Pascual, G., Tempst, P., Rosenfeld, M.G., Glass, C.K. and Kurokawa, R. (2008) Induced ncRNAs allosterically modify RNA-binding proteins in cis to inhibit transcription. *Nature*, **454**, 126-130.
9. Guttman, M. and Rinn, J.L. (2012) Modular regulatory principles of large non-coding RNAs. *Nature*, **482**, 339-346.
10. Rinn, J.L., Kertesz, M., Wang, J.K., Squazzo, S.L., Xu, X., Brugmann, S.A., Goodnough, L.H., Helms, J.A., Farnham, P.J., Segal, E. *et al.* (2007) Functional demarcation of active and silent chromatin domains in human HOX loci by noncoding RNAs. *Cell*, **129**, 1311-1323.
11. Shao, Y., Ye, M., Li, Q., Sun, W., Ye, G., Zhang, X., Yang, Y., Xiao, B. and Guo, J. (2016) LncRNA-RMRP promotes carcinogenesis by acting as a miR-

206 sponge and is used as a novel biomarker for gastric cancer. *Oncotarget*, **7**, 37812-37824.

12. Du, Z., Sun, T., Hacisuleyman, E., Fei, T., Wang, X., Brown, M., Rinn, J.L., Lee, M.G., Chen, Y., Kantoff, P.W. *et al.* (2016) Integrative analyses reveal a long noncoding RNA-mediated sponge regulatory network in prostate cancer. *Nat Commun*, **7**, 10982.
13. Chen, G., Wang, Z., Wang, D., Qiu, C., Liu, M., Chen, X., Zhang, Q., Yan, G. and Cui, Q. (2013) LncRNADisease: a database for long-non-coding RNA-associated diseases. *Nucleic Acids Res*, **41**, D983-986.
14. Huarte, M. (2016) RNA. A lncRNA links genomic variation with celiac disease. *Science*, **352**, 43-44.
15. Li, P., Chen, S., Chen, H., Mo, X., Li, T., Shao, Y., Xiao, B. and Guo, J. (2015) Using circular RNA as a novel type of biomarker in the screening of gastric cancer. *Clin Chim Acta*, **444**, 132-136.
16. Qian, Y., Lu, Y., Rui, C., Qian, Y., Cai, M. and Jia, R. (2016) Potential Significance of Circular RNA in Human Placental Tissue for Patients with Preeclampsia. *Cell Physiol Biochem*, **39**, 1380-1390.
17. Yang, G., Lu, X. and Yuan, L. (2014) LncRNA: a link between RNA and cancer. *Biochim Biophys Acta*, **1839**, 1097-1109.
18. Xie, X., Tang, B., Xiao, Y.F., Xie, R., Li, B.S., Dong, H., Zhou, J.Y. and Yang, S.M. (2016) Long non-coding RNAs in colorectal cancer. *Oncotarget*, **7**, 5226-5239.
19. Han, D., Wang, M., Ma, N., Xu, Y., Jiang, Y. and Gao, X. (2015) Long noncoding RNAs: novel players in colorectal cancer. *Cancer Lett*, **361**, 13-21.
20. Park, N.J., Zhou, H., Elashoff, D., Henson, B.S., Kastratovic, D.A., Abemayor, E. and Wong, D.T. (2009) Salivary microRNA: discovery, characterization, and clinical utility for oral cancer detection. *Clin Cancer Res*, **15**, 5473-5477.
21. Zheng, L.L., Li, J.H., Wu, J., Sun, W.J., Liu, S., Wang, Z.L., Zhou, H., Yang, J.H. and Qu, L.H. (2016) deepBase v2.0: identification, expression, evolution and function of small RNAs, LncRNAs and circular RNAs from deep-sequencing data. *Nucleic Acids Res*, **44**, D196-202.
22. Volders, P.J., Helsens, K., Wang, X., Menten, B., Martens, L., Gevaert, K., Vandesompele, J. and Mestdagh, P. (2013) LNCipedia: a database for annotated human lncRNA transcript sequences and structures. *Nucleic Acids Res*, **41**, D246-251.
23. Glazar, P., Papavasileiou, P. and Rajewsky, N. (2014) circBase: a database for circular RNAs. *RNA*, **20**, 1666-1670.
24. De Smet, R. and Marchal, K. (2010) Advantages and limitations of current network inference methods. *Nat Rev Microbiol*, **8**, 717-729.
25. Wolfe, C.J., Kohane, I.S. and Butte, A.J. (2005) Systematic survey reveals general applicability of "guilt-by-association" within gene coexpression networks. *BMC Bioinformatics*, **6**, 227.
26. Ricano-Ponce, I., Zhernakova, D.V., Deelen, P., Luo, O., Li, X., Isaacs, A., Karjalainen, J., Di Tommaso, J., Borek, Z.A., Zorro, M.M. *et al.* (2016) Refined mapping of autoimmune disease associated genetic variants with gene expression suggests an important role for non-coding RNAs. *J Autoimmun*, **68**, 62-74.
27. D'Haene, E., Jacobs, E.Z., Volders, P.J., De Meyer, T., Menten, B. and Vergult, S. (2016) Identification of long non-coding RNAs involved in neuronal development and intellectual disability. *Sci Rep*, **6**, 28396.

28. Kim, T., Jeon, Y.J., Cui, R., Lee, J.H., Peng, Y., Kim, S.H., Tili, E., Alder, H. and Croce, C.M. (2015) Role of MYC-regulated long noncoding RNAs in cell cycle regulation and tumorigenesis. *J Natl Cancer Inst*, **107**.
29. Fu, M., Huang, G., Zhang, Z., Liu, J., Zhang, Z., Huang, Z., Yu, B. and Meng, F. (2015) Expression profile of long noncoding RNAs in cartilage from knee osteoarthritis patients. *Osteoarthritis Cartilage*, **23**, 423-432.
30. Chen, X., Liu, B., Yang, R., Guo, Y., Li, F., Wang, L. and Hu, H. (2016) Integrated analysis of long non-coding RNAs in human colorectal cancer. *Oncotarget*, **7**, 23897-23908.
31. Lee, S., Kopp, F., Chang, T.C., Sataluri, A., Chen, B., Sivakumar, S., Yu, H., Xie, Y. and Mendell, J.T. (2016) Noncoding RNA NORAD Regulates Genomic Stability by Sequestering PUMILIO Proteins. *Cell*, **164**, 69-80.
32. Kawasaki, Y., Komiya, M., Matsumura, K., Negishi, L., Suda, S., Okuno, M., Yokota, N., Osada, T., Nagashima, T., Hiyoshi, M. *et al.* (2016) MYU, a Target lncRNA for Wnt/c-Myc Signaling, Mediates Induction of CDK6 to Promote Cell Cycle Progression. *Cell Rep*, **16**, 2554-2564.
33. Bailey, T.L., Boden, M., Buske, F.A., Frith, M., Grant, C.E., Clementi, L., Ren, J., Li, W.W. and Noble, W.S. (2009) MEME SUITE: tools for motif discovery and searching. *Nucleic Acids Res*, **37**, W202-208.
34. Grant, C.E., Bailey, T.L. and Noble, W.S. (2011) FIMO: scanning for occurrences of a given motif. *Bioinformatics*, **27**, 1017-1018.
35. Hong, E.L., Sloan, C.A., Chan, E.T., Davidson, J.M., Malladi, V.S., Strattan, J.S., Hitz, B.C., Gabdank, I., Narayanan, A.K., Ho, M. *et al.* (2016) Principles of metadata organization at the ENCODE data coordination center. *Database (Oxford)*, **2016**.
36. Silverman, I.M., Li, F., Alexander, A., Goff, L., Trapnell, C., Rinn, J.L. and Gregory, B.D. (2014) RNase-mediated protein footprint sequencing reveals protein-binding sites throughout the human transcriptome. *Genome Biol*, **15**, R3.
37. Thomson, D.W. and Dinger, M.E. (2016) Endogenous microRNA sponges: evidence and controversy. *Nat Rev Genet*, **17**, 272-283.
38. Lewis, B.P., Burge, C.B. and Bartel, D.P. (2005) Conserved seed pairing, often flanked by adenosines, indicates that thousands of human genes are microRNA targets. *Cell*, **120**, 15-20.
39. Enright, A.J., John, B., Gaul, U., Tuschl, T., Sander, C. and Marks, D.S. (2003) MicroRNA targets in Drosophila. *Genome Biol*, **5**, R1.
40. Kruger, J. and Rehmsmeier, M. (2006) RNAhybrid: microRNA target prediction easy, fast and flexible. *Nucleic Acids Res*, **34**, W451-454.
41. circlncRNAnet GitHub repository. 2017. https://github.com/smw1414/circlncRNAnet

Figure legends

Figure 1. The overall design and the analytic workflow of circlncRNAnet.

Figure 2. Input file formats for circlncRNAnet.

Interface on the webserver for data upload (A). Two files are uploaded prior to data analysis: a gene matrix table (B), which is generated by using featureCounts, and a condition file describing the sample status (C).

Figure 3. Schematic showing example outputs of circlncRNAnet analyses of lncRNA-based networks in colorectal cancer.
After dataset upload, the server executes differential expression and expression correlation analyses. The webserver allows user to select query genes and correlation criteria (A). For an overview of the sequenced transcriptomes, extent of the coordinated expression (B) and overall distribution of non-coding and coding RNA abundance (C) are displayed as summary graphs. As examples of use, co-expression network analysis of a known lncRNA, ELFN1-AS1, and novel lncRNA, XXbac-B476C20.9, was performed using circlncRNAnet. (D) Scatter plot showing the extent of expression correlation between ELFNA-AS1 and one target, MYC.

Figure 4. Additional examples of circlncRNAnet output of lncRNA-based networks in colorectal cancer.
In addition to analyses shown in Figure 3, more options for network interrogation of ncRNA-based regulation can be accessed on the webpage (middle). For instance, heatmap representation of the genes co-expressed with ELFN1-AS1 (Pearson's $|r| > 0.5$) can be outputted (upper left). Pathway analysis of the co-expressed genes on the basis of MSigdb Hallmark pathways (bottom left), and its network depiction of top 3 enriched pathways and the corresponding co-expressed components (bottom right). Circos plot can also be used to illustrate the genome-wide distribution of the top 100 co-expressed genes relative to the location of *XXbac-B476C20.9* (upper right).

Figure 5. Examples of lncRNA-associated molecular components uncovered by circlncRNAnet.
circlncRNAnet may be used to extensively profile the molecular interactome of candidate circRNAs/lncRNAs based on the compiled databases, with ELFN1-AS1 shown as an example in this figure. (A) For the RBP components, the interactome will be outputted in both the table format (top) and network configuration (bottom). (B) Similarly, for the putative miRNA sponge network, predicted ELFN1-AS1-targeting miRNAs are shown in table (top) as well as network (bottom) formats. The web server is also designed to construct the ncRNA-RBP-mRNAs or ncRNA-miRNA-mRNAs regulatory hierarchy. circlncRNAnet delineates co-expressed mRNA genes with mutually shared RBP binding or miRNA targeting sites. Consequently, an intersected gene list is compiled (top) and may be depicted in a two-tier network configuration (bottom).

Table

Click here to download Table table-ver6.pdf

Table 1. Comparative functionalities of available web tools of ncRNAs.

	Tool name	Interface	Both lncRNAs and circRNAs	User data upload option	Expression pattern	Co-expression: gene network	Annotation/ pathway	RBP binding site prediction	miRNA target prediction	Regulatory Network
	circlncRNAnet	Web server	✔	✔	✔	✔	✔	✔	✔	✔
lncRNA	NONCODE	Web database			✔					
lncRNA	LNCipedia	Web database							✔	
lncRNA	ncFANs	Web server		✔		✔	✔			
lncRNA	lncRNAdb	Web database			✔			✔	✔	
lncRNA	LINC	R package		✔		✔	✔			
lncRNA	cogena	R package		✔		✔	✔			
lncRNA	WGCNA	R package		✔		✔				
circRNA	circNet	Web database			✔				✔	✔
circRNA	CIRCpedia	Web database			✔					
circRNA	Circ2Traits	Web database	✔		✔		✔		✔	
circRNA	CircInteractome	Web database						✔	✔	✔
circRNA	DeepBase V2.0	Web database	✔		✔					
circRNA	starBase V2.0	Web database	✔				✔	✔	✔	✔

Click here to download Figure figures-ver2.pdf

Figure 1

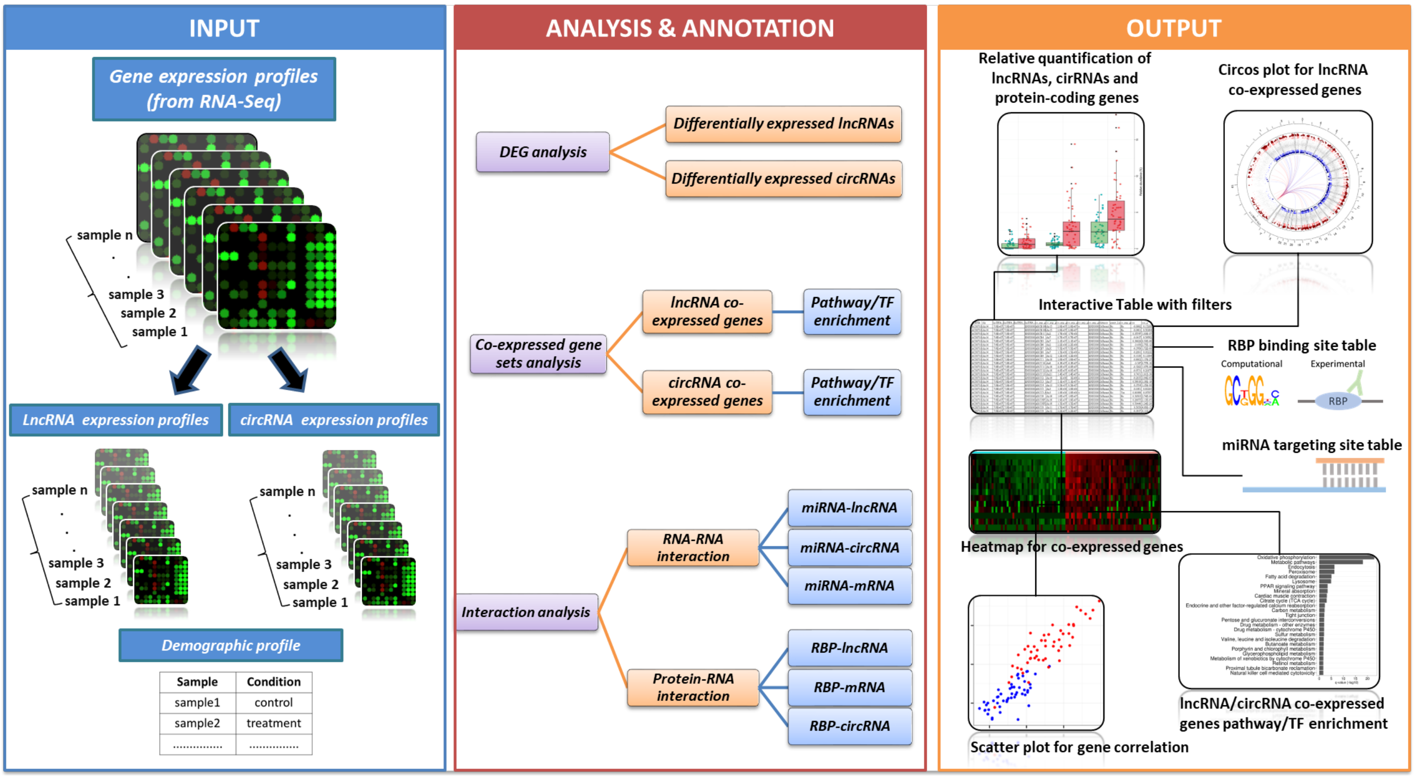

Figure 2

A

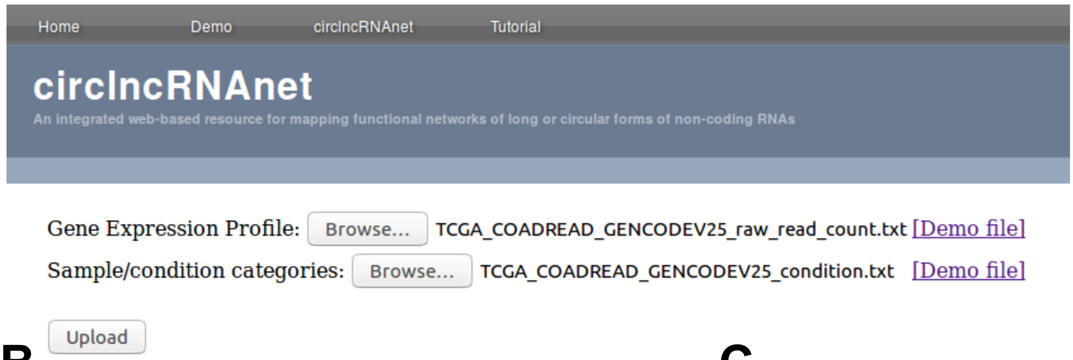

B

Geneid	TCGA-A6-2675-01A-02R-1723-07	TCGA-A6-2675-11A-01R-1723-07											
ENSG00000223972.5	0	0	0	0	0	0	0	0	0	0	0	0	0
ENSG00000227232.5	57	34	27	33	77	20	22	11	33	14	30	25	45
ENSG00000278267.1	2	1	5	3	3	0	3	1	0	1	0	3	0
ENSG00000243485.4	0	0	0	0	0	0	0	0	0	0	0	0	0
ENSG00000237613.2	0	0	0	0	0	0	0	0	0	0	0	0	0
ENSG00000268020.3	0	0	0	0	0	0	0	0	0	0	0	0	0
ENSG00000240361.1	0	0	0	0	0	0	0	0	0	0	0	0	0
ENSG00000186092.4	0	0	0	0	0	0	0	0	0	0	0	0	0
ENSG00000238009.6	0	1	0	0	1	0	0	0	0	0	1	0	0
ENSG00000239945.1	0	0	0	0	0	0	0	0	0	0	0	0	0
ENSG00000233750.3	4	0	14	1	0	1	0	1	0	0	0	0	2
ENSG00000268903.1	63	29	2	46	59	18	0	13	7	8	11	26	34
ENSG00000269981.1	38	11	9	28	19	7	0	8	0	5	4	22	13
ENSG00000239906.1	0	0	0	0	0	0	0	0	0	0	0	0	0

C

TCGA-F4-6704-11A-01R-1839-07	N
TCGA-F4-6704-01A-11R-1839-07	T
TCGA-AZ-6605-11A-01R-1839-07	N
TCGA-AZ-6605-01A-11R-1839-07	T
TCGA-AZ-6603-11A-02R-1839-07	N
TCGA-AZ-6603-01A-11R-1839-07	T
TCGA-AZ-6601-11A-01R-1774-07	N
TCGA-AZ-6601-01A-11R-1774-07	T
TCGA-AZ-6600-11A-01R-1774-07	N
TCGA-AZ-6600-01A-11R-1774-07	T
TCGA-AZ-6599-11A-01R-1774-07	N
TCGA-AZ-6599-01A-11R-1774-07	T
TCGA-AZ-6598-11A-01R-1774-07	N
TCGA-AZ-6598-01A-11R-1774-07	T
TCGA-AH-6643-11A-01R-1830-07	N

Figure 3

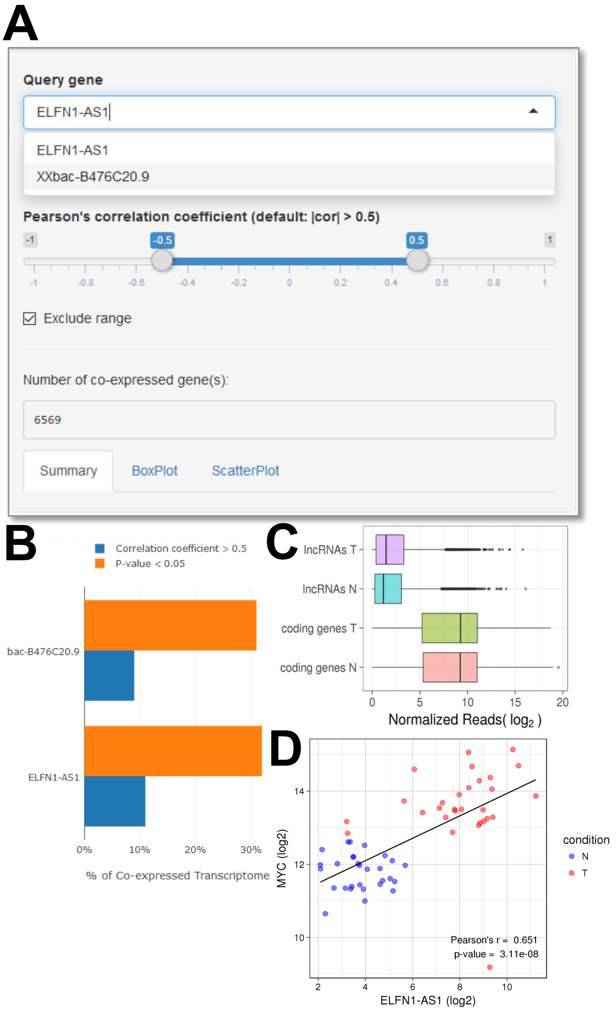
A
Query gene
ELFN1-AS1
ELFN1-AS1
XXbac-B476C20.9
Pearson's correlation coefficient (default: |cor| > 0.5)
-1
-0.5
0.5
1
Exclude range
Number of co-expressed gene(s):
6569
Summary
BoxPlot
ScatterPlot
B
Correlation coefficient > 0.5
P-value < 0.05
bac-B476C20.9
ELFN1-AS1
0%
10%
20%
30%
% of Co-expressed Transcriptome
C
lncRNAs T
lncRNAs N
coding genes T
coding genes N
0
5
10
15
20
Normalized Reads(log_2)
D
MYC (log2)
14
12
10
2
4
6
8
10
ELFN1-AS1 (log2)
Pearson's r = 0.651
p-value = 3.11e-08
condition
N
T

Figure 4

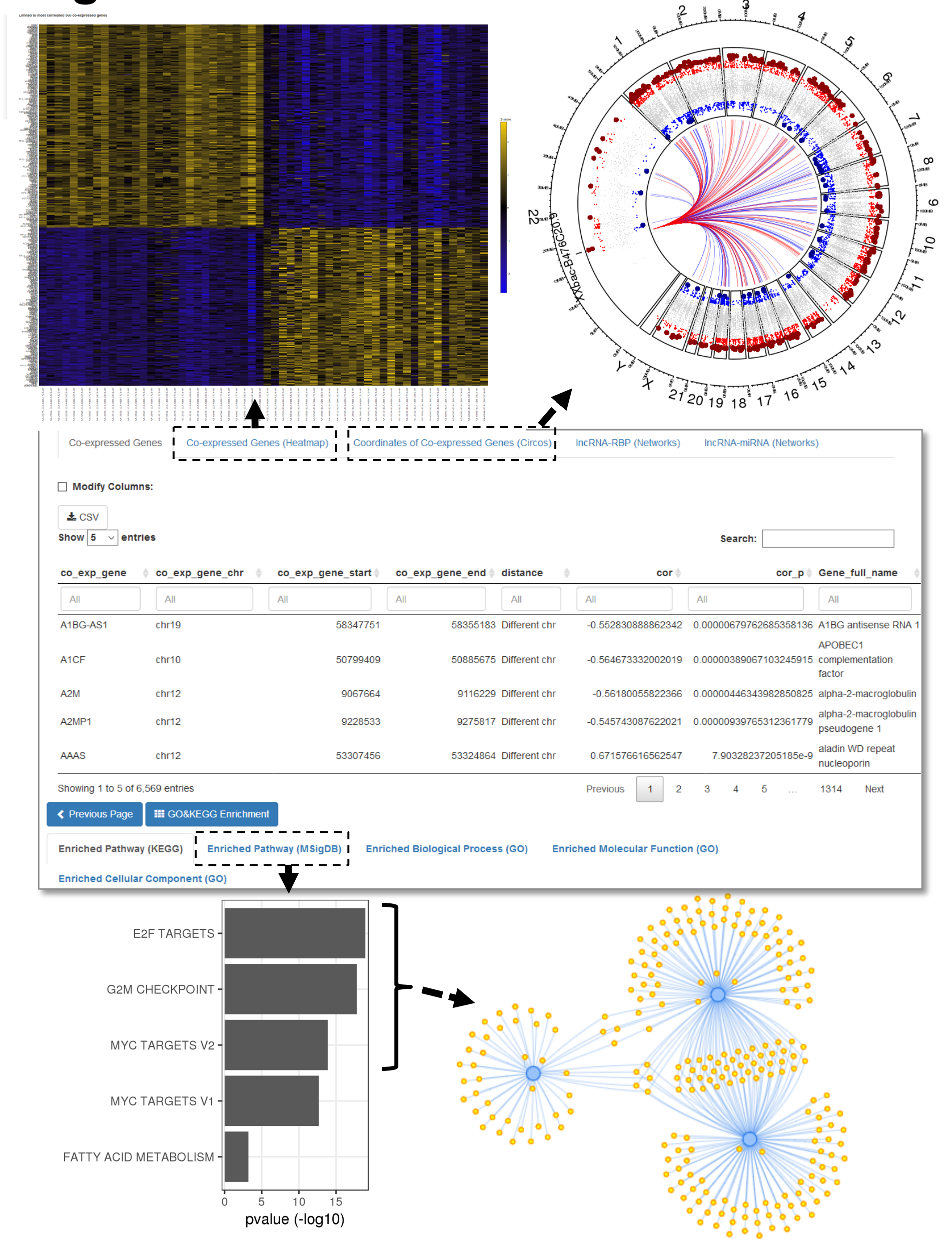
Co-expressed Genes
Co-expressed Genes (Heatmap)
Coordinates of Co-expressed Genes (Circos)
lncRNA-RBP (Networks)
lncRNA-miRNA (Networks)
Modify Columns:
CSV
Show 5 entries
Search:
co_exp_gene | co_exp_gene_chr | co_exp_gene_start | co_exp_gene_end | distance | cor | cor_p | Gene_full_name
A1BG-AS1 | chr19 | 58347751 | 58355183 | Different chr | -0.552830888862342 | 0.00000679762685358136 | A1BG antisense RNA 1
A1CF | chr10 | 50799409 | 50885675 | Different chr | -0.564673332002019 | 0.00000389067103245915 | APOBEC1 complementation factor
A2M | chr12 | 9067664 | 9116229 | Different chr | -0.56180055822366 | 0.00000446343982850825 | alpha-2-macroglobulin
A2MP1 | chr12 | 9228533 | 9275817 | Different chr | -0.545743087622021 | 0.00000939765312361779 | alpha-2-macroglobulin pseudogene 1
AAAS | chr12 | 53307456 | 53324864 | Different chr | 0.671576616562547 | 7.90328237205185e-9 | aladin WD repeat nucleoporin
Showing 1 to 5 of 6,569 entries
Previous 1 2 3 4 5 ... 1314 Next
Previous Page
GO&KEGG Enrichment
Enriched Pathway (KEGG)
Enriched Pathway (MSigDB)
Enriched Biological Process (GO)
Enriched Molecular Function (GO)
Enriched Cellular Component (GO)
E2F TARGETS
G2M CHECKPOINT
MYC TARGETS V2
MYC TARGETS V1
FATTY ACID METABOLISM
0 5 10 15
pvalue (-log10)

Figure 5

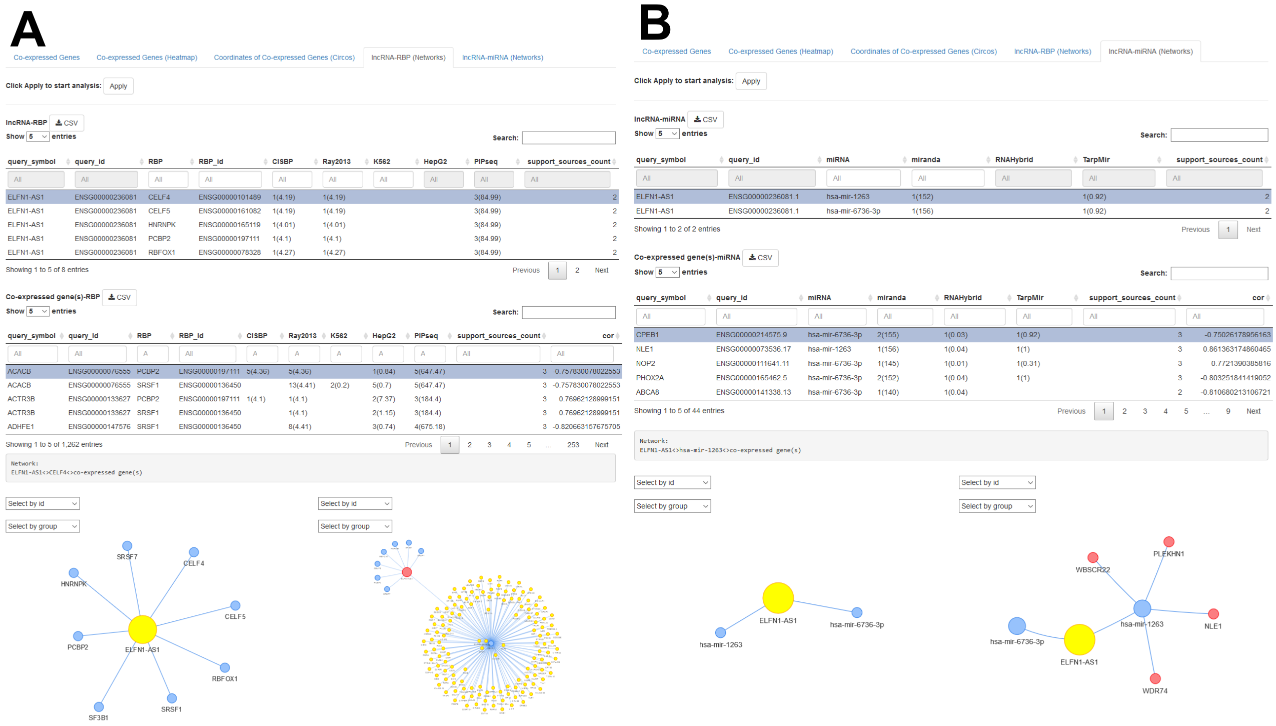
A
Co-expressed Genes
Co-expressed Genes (Heatmap)
Coordinates of Co-expressed Genes (Circos)
lncRNA-RBP (Networks)
lncRNA-miRNA (Networks)
Click Apply to start analysis:
Apply
lncRNA-RBP
CSV
Show 5 entries
Search:
query_symbol query_id RBP RBP_id CISBP Ray2013 K562 HepG2 PIPseq support_sources_count
ELFN1-AS1 ENSG00000236081 CELF4 ENSG00000101489 1(4.19) 1(4.19) 3(84.99) 2
ELFN1-AS1 ENSG00000236081 CELF5 ENSG00000161082 1(4.19) 1(4.19) 3(84.99) 2
ELFN1-AS1 ENSG00000236081 HNRNPK ENSG00000165119 1(4.01) 1(4.01) 3(84.99) 2
ELFN1-AS1 ENSG00000236081 PCBP2 ENSG00000197111 1(4.1) 1(4.1) 3(84.99) 2
ELFN1-AS1 ENSG00000236081 RBFOX1 ENSG00000078328 1(4.27) 1(4.27) 3(84.99) 2
Showing 1 to 5 of 8 entries
Previous 1 2 Next
Co-expressed gene(s)-RBP
query_symbol query_id RBP RBP_id CISBP Ray2013 K562 HepG2 PIPseq support_sources_count cor
ACACB ENSG00000076555 PCBP2 ENSG00000197111 5(4.36) 5(4.36) 1(0.84) 5(647.47) 3 -0.757830078022553
ACACB ENSG00000076555 SRSF1 ENSG00000136450 13(4.41) 2(0.2) 5(0.7) 5(647.47) 3 -0.757830078022553
ACTR3B ENSG00000133627 PCBP2 ENSG00000197111 1(4.1) 1(4.1) 2(7.37) 3(184.4) 3 0.76962128999151
ACTR3B ENSG00000133627 SRSF1 ENSG00000136450 1(4.1) 2(1.15) 3(184.4) 3 0.76962128999151
ADHFE1 ENSG00000147576 SRSF1 ENSG00000136450 8(4.41) 3(0.74) 4(675.18) 3 -0.820663157675705
Showing 1 to 5 of 1,262 entries
Previous 1 2 3 4 5 ... 253 Next
Network:
ELFN1-AS1<>CELF4<>co-expressed gene(s)
Select by id
Select by group
SRSF7
CELF4
HNRNPK
CELF5
PCBP2
ELFN1-AS1
RBFOX1
SRSF1
SF3B1
B
Co-expressed Genes
Co-expressed Genes (Heatmap)
Coordinates of Co-expressed Genes (Circos)
lncRNA-RBP (Networks)
lncRNA-miRNA (Networks)
Click Apply to start analysis:
Apply
lncRNA-miRNA
CSV
Show 5 entries
Search:
query_symbol query_id miRNA miranda RNAHybrid TarpMir support_sources_count
ELFN1-AS1 ENSG00000236081.1 hsa-mir-1263 1(152) 1(0.92) 2
ELFN1-AS1 ENSG00000236081.1 hsa-mir-6736-3p 1(156) 1(0.92) 2
Showing 1 to 2 of 2 entries
Previous 1 Next
Co-expressed gene(s)-miRNA
query_symbol query_id miRNA miranda RNAHybrid TarpMir support_sources_count cor
CPEB1 ENSG00000214575.9 hsa-mir-6736-3p 2(155) 1(0.03) 1(0.92) 3 -0.75026178956163
NLE1 ENSG00000073536.17 hsa-mir-1263 1(156) 1(0.04) 1(1) 3 0.861363174860465
NOP2 ENSG00000111641.11 hsa-mir-6736-3p 1(145) 1(0.01) 1(0.31) 3 0.7721390385816
PHOX2A ENSG00000165462.5 hsa-mir-6736-3p 2(152) 1(0.04) 1(1) 3 -0.803251841419052
ABCA8 ENSG00000141338.13 hsa-mir-6736-3p 1(140) 1(0.04) 2 -0.810680213106721
Showing 1 to 5 of 44 entries
Previous 1 2 3 4 5 ... 9 Next
Network:
ELFN1-AS1<>hsa-mir-1263<>co-expressed gene(s)
Select by id
Select by group
ELFN1-AS1
hsa-mir-1263
hsa-mir-6736-3p
PLEKHN1
WBSCR22
NLE1
WDR74
