# Supplementary material for: circlncRNAnet: an integrated web-based resource for mapping functional networks of long or circular forms of noncoding RNAs
Source: Gigascience. 2017 Nov 29;7(1):gix118. doi: 10.1093/gigascience/gix118 (PMC5765557; doi:10.1093/gigascience/gix118)

GigaScience

circlncRNAnet: An integrated web-based resource for mapping functional networks of long or circular forms of non-coding RNAs

--Manuscript Draft--

**Manuscript Number:**	GIGA-D-17-00121R1
**Full Title:**	circlncRNAnet: An integrated web-based resource for mapping functional networks of long or circular forms of non-coding RNAs
**Article Type:**	Technical Note
**Funding Information:**	Ministry of Science and Technology, Taiwan (TW): Dr. Hsuan Liu<br>Dr. Po-Jung Huang<br>Dr. Bertrand Chin-Ming Tan<br>Chang Gung Memorial Hospital, Linkou: Dr. Hsuan Liu<br>Dr. Wen-Sy Tsai<br>Dr. Bertrand Chin-Ming Tan
**Abstract:**	Background<br>Despite their lack of protein-coding potential, lncRNAs and circRNAs have emerged as key determinant in gene regulation, acting to fine-tune transcriptional and signaling output. These non-coding RNA transcripts are known to affect expression of messenger RNAs (mRNAs) via epigenetic and post-transcriptional regulation. Given their widespread target spectrum as well as extensive modes of action, a complete understanding of their biological relevance will depend on integrative analyses of systems data at various levels.<br><br>Findings<br>While a handful of publicly available databases have been reported, existing tools do not fully capture from a network perspective the functional implications of lncRNAs or circRNAs of interest. Through an integrated and streamlined design, circlncRNAnet is aimed to broaden the understanding of ncRNA candidates by testing in silico several hypotheses of ncRNA-based functions on the basis of large-scale RNA-seq data. This web server is implemented with several features representing advances in the bioinformatics of ncRNAs: 1) a flexible framework that accepts and processes user-defined NGS-based expression data; 2) multiple analytic modules that assign and productively assess the regulatory networks of user-selected ncRNAs by cross-referencing extensively curated databases; 3) an all-purpose, information-rich workflow design that is tailored to all types of ncRNAs. Outputs on expression profiles, co-expression networks & pathways, and molecular interactomes, are dynamically and interactively displayed according to user-defined criteria.<br><br>Conclusions<br>In short, users may apply circlncRNAnet to obtain, in real time, multiple lines of functionally relevant information on the circRNAs/lncRNAs of their interest. In summary, circlncRNAnet is the first of its kind in the regulatory RNA research field, providing a "one-stop" resource for in-depth analyses of ncRNA biology. circlncRNAnet is freely available at http://app.cgu.edu.tw/circlnc/.
**Corresponding Author:**	Bertrand Tan, Ph.D.<br>Chang Gung University<br>Tao-Yuan, TAIWAN
**Corresponding Author Secondary Information:**	
**Corresponding Author's Institution:**	Chang Gung University
**Corresponding Author's Secondary Institution:**	
**First Author:**	Shao-Min Wu
**First Author Secondary Information:**	

Powered by Editorial Manager® and ProduXion Manager® from Aries Systems Corporation

<table>
<tr><td>Order of Authors:</td><td>Shao-Min Wu</td></tr>
<tr><td></td><td>Hsuan Liu</td></tr>
<tr><td></td><td>Po-Jung Huang</td></tr>
<tr><td></td><td>Ian Yi-Feng Chang</td></tr>
<tr><td></td><td>Chi-Ching Lee</td></tr>
<tr><td></td><td>Chia-Yu Yang</td></tr>
<tr><td></td><td>Wen-Sy Tsai</td></tr>
<tr><td></td><td>Bertrand Chin-Ming Tan, Ph.D.</td></tr>
<tr><td>Order of Authors Secondary Information:</td><td></td></tr>
<tr><td>Response to Reviewers:</td><td>Point-by-point response to referees comments (MS ID# GIGA-D-17-00121_R1)
(>: our responses)
(Modifications and the inclusion of new data have been marked in red in the revised manuscript and summarized in List of amendments to manuscript)

Reviewer #1: Overall the tool implements a wide variety of functionality and appears to function as described. I believe this will be a useful to biologists studying lncRNAs. There are a few areas I believe should be addressed before this paper is ready for publication.

1) Quite a large number of sentences seem to be missing citations.
Ex:
"Both lncRNAs and circRNAs have been found to harbor microRNA response elements (MREs) and potentially act as "miRNA sponges" that sequester these endogenous small RNAs"
I guess you are intending for the citations in the following sentence to cover this sentence as well?

>Thank you for pointing this out. The citations in the following sentence were indeed intended for this sentence. We have moved the citations to the more appropriate location (p. 3, lines 31-32). We also provided more references according to reviewer’s comments below (points #2 and #3).

1) At the moment I believe the writing and formatting is the biggest issue with this paper. These make the article difficult to follow in some sections.

>We appreciate the valuable suggestions on writing. All the suggestions have been addressed accordingly. To improve the overall quality of writing, we have also thoroughly reviewed the text and made all the necessary changes.

1a) The article is verbose, making it difficult to follow.
Ex:
"At the mechanistic level, regardless of the forms, these non-coding RNA transcripts have been known to impact the expression of messenger RNAs (mRNA) via epigenetic and post-transcriptional regulation."

This sentence could be shortened to something like this.
"Non-coding RNAs are known to affect messenger RNA (mRNA) expression via epigenetic and post-transcriptional regulation"

>The suggested modification is now incorporated in the Abstract (page 2, line 5).

1b) The word usage is a bit strange in some places.
Ex:
"To circumvent this problem, we have implemented an integrative bioinformatics approach to approach to examine in silico the cellular roles of lncRNAs by mapping their functional networks."

Solve is more appropriate instead of circumvent. Circumvent sounds like you are finding a way around the problem without fixing it. Note this sentence is also verbose. I</td></tr>
</table>

Powered by Editorial Manager® and ProduXion Manager® from Aries Systems Corporation

think reducing the verbosity and complexity of the language in the paper will help solve word usage problems.

>Thank you for the suggestion. We have made the corresponding adjustment (page 4, line 9). In addition, we have modified this sentence to improve clarity: “To solve this problem, we have implemented an integrative bioinformatics approach to examine in silico the functional networks of ncRNAs.”

1c) Use more nouns and fewer pronouns.
>We have addressed this issue by making the following changes:
“It” is changed to “The web server” (page 2, line 17).
“They” is changed to “lncRNAs” (page 3, line 14).

2) Many researchers now consider circRNAs to be lncRNAs. Maybe add something like "… and in fact circRNAs are considered to be a class of lncRNAs by many researchers.. (1)" to "Despite their differences in structure and biosynthesis steps, lncRNAs and circRNAs are much more common in terms of their roles and mechanism in gene regulation"
1. Quinn, Jeffrey J., and Howard Y. Chang. "Unique features of long non-coding RNA biogenesis and function." Nature Reviews Genetics 17.1 (2016): 47-62.

>Thank you for the insightful comments and important references (both points #2 and #3). There are indeed certain similarity and distinctions between these two types of ncRNAs. We have now revised the descriptions accordingly. The above publication is cited as new reference #3 in page 3, line 21

3) The evidence lncRNAs acting as miRNA sponges is much stronger than the evidence for circRNAs acting as miRNA sponges. I recommend adding a sentence to the effect of "Although the evidence for lncRNA miRNA sponges is much stronger than circRNA sponges (1). " after "Both lncRNAs and circRNAs have been found to harbor microRNA response elements (MREs) and potentially act as "miRNA sponges" that sequester these endogenous small RNAs"

1. Militello, Giuseppe, et al. "Screening and validation of lncRNAs and circRNAs as miRNA sponges." Briefings in Bioinformatics (2016): bbw053.
2. Boeckel, Jes-Niels, et al. "Identification and characterization of hypoxia-regulated endothelial circular RNA." Circulation research (2015): CIRCRESAHA-115.

>We agree with the reviewer that, due to the unique structure and abundance, lncRNAs may confer a much stronger effect on miRNA sponging. These two references are added as: “… although the evidence for lncRNA miRNA sponges is much stronger than circRNA sponges [13, 14].” (page 3, lines 32-33).

4) I recommend increasing the quality of the github repository by adding comments to your code and enough documentation that a user could set up their own instance of your server.

>Thank you for the kind reminder. We have now included additional information on how the modules were installed and connected. We also provided on GitHub instructions on running our pipeline in local mode.

Reviewer #2: The pipeline and associated website proposed by the authors is of interest for researchers wanting to better characterize one or several non-coding RNAs.

[My first major criticism is that the the website was not accessible on July 29th and August 1st which prevented me to assess the proposed tool. / Editors' note, update 13/8: The reviewer checked again after sending his report, and could meanwhile assess the homepage.]

>We apologize for the temporary shutdown of the web server. There was an unexpected power outage at the building where the server as housed.

The manuscript contains very little detail on how the analyses are performed, I had to

check the different scripts available on the GitHub repository was able to find a github repository to understand the analyses performed (differential expression, co-expression analysis). Additionally there is no list of the packages called by the program, nor does the manuscript highlight the species and references supported. Furthermore there is no information regarding the parameters used to run the different programs (miRanda, RNAHybrid ...). The authors need to significantly develop this, it is nice to have examples of the different analyses the pipeline enables but it is of utmost importance that the reader gains a good grasp of the tools and analyses performed by the pipeline and the parameters used.

>We thank the reviewer for this important reminder. These lines of information were available at the time of submission but omitted from the text for space conservation. As suggested by the reviewer, these details on the analyses are now incorporated in the manuscript: differential expression (DESeq2), page 5, lines 3; co-expression analysis (WGCNA), page 5, lines 20-22. Furthermore, we listed in two new tables the software tools (Table2) and databases (Table 3) called by our server. In Table 3 with software tools, we also provided the parameters used to run these programs. We incorporated this information in the “Output summary” section of the Results (page 4, lines 36-37).

Minor comments the authors mention that the users can define the gene list after differential expression using the fold changes and p-values. Are they using the p-value or the adjust p-value? From the scripts available on github (deseq2_annotation.r) it appears that the column used is the uncorrected p-value which implies a list of differentially expressed genes heavily contaminated by false positives.

>Thank you for this reminder. “Adjusted p-values” (p-adjusted or padj on the web site) are now used as the criterion for distinguishing differential expression.

It would be very useful to the user if the authors could guide them in the choice of the Pearson's coefficient of correlation, such as using a randomization approach on the submitted sets to identify the thresholds.

>Selection of appropriate threshold for correlation is indeed important to subsequent network analyses. Randomization approach for threshold selection is now incorporated at this step of data analysis (500 iterations of randomized Pearson correlations between target ncRNA and 5,000 randomly selected mRNAs). As a guide to the users, the server will display a composite histogram showing the overall distribution of correlation coefficients calculated for all the ncRNA-mRNAs pairs, superimposed with the results from randomized correlation tests. This new function is now described as Figure 3E on page 5, lines 25-31.

List of amendments to manuscript (MS ID# GIGA-D-17-00121_R1)
Referees’ comments have been addressed in detail in the “Point-by-point response to referees comments”. Modifications and the inclusion of new data have been marked in red in the revised manuscript and summarized by the following points:

Abstract:
P2. Minor changes in wording were made in response to reviewer’s suggestions (lines 5 and 17).

Introduction:
P3. Two new sentences and corresponding references (#3, #13 and #14) were incorporated in the second paragraph (lines 20-21 and 32-33).

Results and Methods:
P4. Two new tables (Tables 2 and 3) and corresponding descriptions were added in the “Output summary” section (lines 36-37).
P4-5. Descriptions for several analyses, such as differential expression (p. 5, line 3) expression correlation (p. 5, lines 20-22), as well as randomized correlation test (p. 5, lines 25-31), were added in the “Analytic module #1: coding–non-coding co-expression (CNC) network profiling” section.

Availability of supporting data:

	P9. For the convenience of prospective users, we also provided on GitHub instructions on running our pipeline in local mode. This statement is added in lines 10-12.<br><br>GitHub repository:<br>We provided GitHub additional details on how the modules are executed and also instructions on running our pipeline in local mode.<br><br>Figures and Legends:<br>Figure 3E: An exemplary histogram of randomized test results, together with the corresponding legends, were incorporated.
**Additional Information:**	
**Question**	**Response**
Are you submitting this manuscript to a special series or article collection?	No
**Experimental design and statistics**<br><br>Full details of the experimental design and statistical methods used should be given in the Methods section, as detailed in our Minimum Standards Reporting Checklist. Information essential to interpreting the data presented should be made available in the figure legends.<br><br>Have you included all the information requested in your manuscript?	Yes
**Resources**<br><br>A description of all resources used, including antibodies, cell lines, animals and software tools, with enough information to allow them to be uniquely identified, should be included in the Methods section. Authors are strongly encouraged to cite Research Resource Identifiers (RRIDs) for antibodies, model organisms and tools, where possible.<br><br>Have you included the information requested as detailed in our Minimum Standards Reporting Checklist?	Yes
**Availability of data and materials**<br><br>All datasets and code on which the conclusions of the paper rely must be either included in your submission or deposited in publicly available repositories (where available and ethically appropriate), referencing such data using a unique identifier in the references and in	Yes

Powered by Editorial Manager® and ProduXion Manager® from Aries Systems Corporation

the “Availability of Data and Materials” section of your manuscript.<br><br>Have you have met the above requirement as detailed in our Minimum Standards Reporting Checklist?	

Powered by Editorial Manager® and ProduXion Manager® from Aries Systems Corporation

Manuscript Click here to download Manuscript GIGA-D-17-00121R1_211117ED.doc

circlncRNAnet: An integrated web-based resource for mapping functional networks of long or circular forms of non-coding RNAs

Shao-Min Wu[1,*], Hsuan Liu[1,2,3,4,*], Po-Jung Huang[3,5,6,*], Ian Yi-Feng Chang[3], Chi-Ching Lee[7], Chia-Yu Yang[1,3,4,8], Wen-Sy Tsai[4], Bertrand Chin-Ming Tan[1,3,5,9]

[1]Graduate Institute of Biomedical Sciences, College of Medicine, Chang Gung University, Guishan, Taoyuan, Taiwan; [2]Department of Cell and Molecular Biology, College of Medicine, Chang Gung University, Guishan, Taoyuan, Taiwan; [3]Molecular Medicine Research Center, Chang Gung University, Guishan, Taoyuan, Taiwan; [4]Division of Colon and Rectal Surgery, Department of Surgery, Chang Gung Memorial Hospital, Linkou, Taiwan; [5]Department of Biomedical Sciences, College of Medicine, Chang Gung University, Guishan, Taoyuan, Taiwan; [6]Genomic Medicine Research Core Laboratory, Chang Gung Memorial Hospital, Linkou, Taiwan; [7]Department of Computer Science and Information Engineering, College of Engineering, Chang Gung University, Guishan, Taoyuan, Taiwan; [8]Department of Microbiology and Immunology, College of Medicine, Chang Gung University, Guishan, Taoyuan, Taiwan; [9]Department of Neurosurgery, Linkou Medical Center, Chang Gung Memorial Hospital, Linkou, Taiwan

*These authors contributed equally to this work.

Correspondence to:

Bertrand Chin-Ming Tan, Graduate Institute of Biomedical Sciences and Department of Biomedical Sciences, College of Medicine, Chang Gung University, 259 Wenhua 1st Road, Guishan, Taoyuan 333, Taiwan, ROC. Tel.: +886-3-211-8800; Fax: +866-3-211-8700; E-mail: btan@mail.cgu.edu.tw

Wen-Sy Tsai, Division of Colon and Rectal Surgery, Department of Surgery, Chang Gung Memorial Hospital, Linkou, Taiwan, ROC. E-mail: wensy@adm.cgmh.org.tw

Abstract

Background

Despite their lack of protein-coding potential, lncRNAs and circRNAs have emerged as key determinants in gene regulation, acting to fine-tune transcriptional and signaling output. These non-coding RNA transcripts are known to affect expression of messenger RNAs (mRNAs) via epigenetic and post-transcriptional regulation. Given their widespread target spectrum as well as extensive modes of action, a complete understanding of their biological relevance will depend on integrative analyses of systems data at various levels.

Findings

While a handful of publicly available databases have been reported, existing tools do not fully capture, from a network perspective, the functional implications of lncRNAs or circRNAs of interest. Through an integrated and streamlined design, circlncRNAnet aims to broaden the understanding of ncRNA candidates by testing *in silico* several hypotheses of ncRNA-based functions, on the basis of large-scale RNA-seq data. This web server is implemented with several features representing advances in the bioinformatics of ncRNAs: 1) a flexible framework that accepts and processes user-defined NGS-based expression data; 2) multiple analytic modules that assign and productively assess the regulatory networks of user-selected ncRNAs by cross-referencing extensively curated databases; 3) an all-purpose, information-rich workflow design that is tailored to all types of ncRNAs. Outputs on expression profiles, co-expression networks & pathways, and molecular interactomes, are dynamically and interactively displayed according to user-defined criteria.

Conclusions

In short, users may apply circlncRNAnet to obtain, in real time, multiple lines of functionally relevant information on circRNAs/lncRNAs of their interest. In summary, circlncRNAnet provides a “one-stop” resource for in-depth analyses of ncRNA biology. circlncRNAnet is freely available at http://app.cgu.edu.tw/circlnc/.

Keywords

lncRNAs – circRNAs – co-expression network – molecular interactome

Introduction

Only 1% of the human genome encodes proteins. In contrast, 70% to 90% of the genome can actually be transcribed at some point during development, generating a large transcriptome of non-coding RNAs (ncRNA), part of which ultimately yield definite short or long RNAs with limited protein-coding capacity [1]. In recent years, deep sequencing technologies have unraveled the non-coding constituents of the transcriptome, most notably lncRNAs and circRNAs. Despite the lack of protein-coding potential, these once uncharted parts have emerged as a key determinant in gene regulation, acting as critical switches that fine-tune transcriptional and signaling output [2, 3].

Distinct from small non-coding RNAs such as microRNAs and snRNAs, long non-coding RNAs (lncRNAs) are RNA molecules with a length of more than 200 nucleotides lacking a detectable open reading frame [4]. lncRNAs are usually transcribed by RNA polymerase II and exhibit known attributes of messenger RNAs, such as post-transcriptional processing. Circular RNAs (circRNAs) are a more recently discovered class of non-coding RNAs, which is defined not by length but rather the unique structure of covalently closed circularity [5, 6]. Despite their differences in structure and biosynthesis steps, lncRNAs and circRNAs are much more common in terms of their roles and mechanisms in gene regulation, and in fact circRNAs are considered to be a class of lncRNAs by many researchers [3]. Even in the absence of protein products, these RNA molecules have been found to associate with distinct cellular compartments or components, and may act *in cis* or *trans* in target gene regulation, [7-10]: At the epigenetic and transcriptional level, lncRNAs are known to interact with transcriptional activators or repressors and consequently impact transcriptional efficiency. By binding with chromatin-modifying factors, lncRNAs could also serve as guide or scaffold that controls the epigenetic status. At the post-transcription level, lncRNAs may bind to target RNAs and alter transcript structure, splicing pattern and stability. Both lncRNAs and circRNAs have been found to harbor microRNA response elements (MREs) and potentially act as "miRNA sponges" that sequester these endogenous small RNAs [8, 11, 12], although the evidence for lncRNA miRNA sponges is much stronger than circRNA sponges [13, 14]. These ncRNAs are therefore part of the competing endogenous RNA (ceRNA) network with the potential to alter the miRNA-targeted mRNA expression. Another mode of regulation exerted by lncRNAs is their association with RNA-binding proteins. Similar to the ceRNA scenario, this molecular interaction may impact the localization, and thus activity, of these gene regulators. Finally, in line with their critical roles as gene regulators, both circRNAs and lncRNAs exhibit unique expression profiles in various human cancers, suggestive of a correlation with disease progression and possibly its value as predictor of patient outcome [15-19]. Delineation of these transcriptomic networks therefore is of importance in understanding ncRNAs and associated biological processes and may shed new light on diseases and possibly new avenues of therapeutic interventions [20-22].

Despite the enormous number of lncRNAs (~15,000) annotated by GENCODE [23], functional understanding of the lncRNAs remains largely limited. While large-scale sequencing studies have become a standard approach for identifying candidate circRNAs/lncRNAs with significant expression alteration in certain cellular states, there may not be sufficient information in the literature to warrant further functional interrogation. Moreover, given the potentially widespread target spectrum of these

ncRNAs as well as their extensive modes of action, a complete understanding of their biological relevance will depend on integrative analyses of systems data at various levels [24]. While a handful of publicly available databases have been reported (Table 1), they are quite limited in the scope of reference data and analytic modules, relying on existing datasets in public archives and annotating pre-selected regulatory features of ncRNAs. Thus, existing tools do not fully capture, from a network perspective, the functional implications of lncRNAs or circRNAs of interest. To solve this problem, we have implemented an integrative bioinformatics approach to examine *in silico* the functional networks of ncRNAs. The overall design and the analytic workflow of this first "one-stop" web server tool for exploring the ncRNA biology are depicted in Figure 1.

[Insert Table 1. here]

Results and Methods

Data input

To start, there are two separate upload pages for "lncRNA" and "circRNA", to meet distinct analytic requirements of these two types of molecules (Fig. 2A). Users may upload tab-delimited text files that contain 1) expression matrix data of RNA-seq raw read counts, which are generated by using featureCounts [38] (Fig. 2B) and 2) sample/condition categories (Fig. 2C), respectively into "Gene Expression Profile" and "Demographic Information" on the webpage. For circRNA analyses, circRNA read counts, as quantified by KNIFE [39], should be additionally provided in a separate file. Procedures for processing the datasets into the appropriate format are outlined in the tutorial page on the web server (http://app.cgu.edu.tw/circlnc/). For demonstration of use, two test datasets derived from publicly available RNA-seq data are included in the web server: The Cancer Genome Atlas (TCGA) data on colon and rectal adenocarcinoma (COAD and READ) (for lncRNA) and the Encyclopedia of DNA Elements (ENCODE) data on esophagus and sigmoid colon (for circRNA) [40, 41].

Output summary

After the successful submission of a job, processing statuses, file format conversion, co-expression analysis, interactome networking, and report generation, are displayed using a dynamic progress indicator. Computational tools and databases employed in this study are listed in Tables 2 and 3, respectively, which also outline the parameters used to carry out the corresponding analyses. The output section of tutorial page (http://app.cgu.edu.tw/circlnc/) shows the standard output of circlncRNAnet based on the demonstration datasets. The standard output is represented by dynamic tables and charts, including bar and box plots, scatter plot, circos plot, heatmap, and network plots. Also included in the table is annotation information of the coding and non-coding genes, such as genome location, distance from query lncRNA or circRNA, lncRNA ID (ENCODE), coding potential [26], circRNA ID according to circBase [42], circRNA (or host gene) splicing structure.

[Insert Table 2. here]

[Insert Table 3. here]

Analytic module #1: coding–non-coding co-expression (CNC) network profiling

After the upload, the server will first execute the differential expression analysis by using the R package DESeq2 [45]. The interactive interface allows users to define the candidate gene list by fold changes and *p*-value. Moreover, to inspect the expression distance between samples, principal components analysis (PCA) was implemented in our analysis pipeline.

Several known functional attributes of circRNAs/lncRNAs were taken into account when constructing this web server: First, we adopted the gene co-expression analysis, which is based on the concept of "guilt by association" – assuming that genes exhibiting analogous expression patterns may be involved in similar biological pathways, functions of unknown genes may be inferred *a priori* from the co-expressed, functionally known genes [57]. To this end, Wolfe et al. developed a method to demonstrate that co-expression with biologically defined modules may serve as a basis for characterizing the function of unknown genes [58]. Ricano-Ponce et al. also used co-expression analysis to deduce the function of lncRNAs with expression quantitative trait loci (eQTLs) effects [59]. The combined use of co-expression analysis and GSEA (Gene Set Enrichment Analysis) has been demonstrated to identify lncRNAs putatively involved in neuronal development [60]. To implement this co-expression analysis in circlncRNAnet, we used the R package WGCNA [31] to calculate the Pearson correlation coefficients of selected differentially-expressed circRNAs/lncRNAs expression against all genes in the user-uploaded samples (Fig. 3A). For an overview of the sequenced transcriptomes, extent of the coordinated expression (Fig. 3B) and overall distribution of non-coding and coding RNA abundance (Fig. 3C) can be displayed as summary graphs. To provide users with a guide in the selection of relevant criteria for expression correlation, the server displays a composite histogram showing the overall distribution of correlation coefficients calculated for all the ncRNA-mRNAs pairs, superimposed with the results from randomized correlation tests (500 iterations of randomized Pearson correlations between target ncRNA and 5,000 randomly selected mRNAs). The highly correlated genes (based on user-defined Pearson's correlation) will also be subjected to pathway enrichment analysis (Fig. 4). The identity and enriched terms of the co-expression networks will be provided to facilitate further functional deduction of ncRNAs candidates.

As a proof of principle, we applied our analytic pipeline to a known example of cancer-associated lncRNAs, *ELFN1-AS1*. Kim *et al.* recently reported that MYC-regulated lncRNA *MYCLo-2* (also known as *ELFN1-AS1*) represses *CDKN2B* transcription coordinately with hnRNPK [61]. To demonstrate the utility of circlncRNAnet, we queried the functional network of *ELFN1-AS1*. We used The Cancer Genome Atlas (TCGA) data on COAD and READ and paired normal samples as the reference expression datasets. Co-expression gene network analysis for *ELFN1-AS1* may be done on the basis of the differentially expressed gene list and outputted according to user-defined criteria (Fig. 4, middle panel). To further visualize overall expression profiles of *ELFN1-AS1* co-expressed genes, "heatmap" may be used to display up to 500 of most correlated genes (ranked by absolute *r* value) (Fig. 4, upper left panel). Pair-wise expression correlation between the ncRNA and co-expressed mRNA genes is also possible. For instance, as *ELFN1-AS1* is a known transcriptional target of MYC, users may compare the expression patterns between *ELFN1-AS1* and *MYC* in the TCGA data. This is done through "Scatter plot" and enter "MYC" in the "Co-expressed gene" box (Fig. 3D). Next, pathway analysis of genes co-expressed

with *ELFN1-AS1*, the "GO & KEGG Enrichment" functionality is available, in which the "Enriched pathway (MSigDB)" will output top enriched pathways, together with a network representation of the components. In the case of *ELFN1-AS1*, MYC TARGETS V1 and MYC TARGETS V2 are shown as two of the top pathways, consistently with the previous findings (Fig. 4, lower panels).

In addition, we used another novel lncRNA as an example of our analytic approach. *XXbac-B476C20.9* was downregulated in the colorectal cancer, and higher expression of *XXbac-B476C20.9* exhibited better survival expectancy, hinting at a tumor-suppressive role (data not shown). By using Pearson correlation analysis, we identified hundreds of genes that exhibit significant co-expression with this lncRNA (data not shown). By analyzing the chromosome distribution of *XXbac-B476C20.9* co-expressed genes, we did not see particular enrichment in chromosome 22 (where *XXbac-B476C20.9* locates) (Fig. 4, upper right panel), indicating that this lncRNA may not exert expression regulation in a cis manner.

Correlated expression may also be attributed to the functional interaction of the circRNAs/lncRNAs with particular transcription factor (TF) networks. Indeed, previous studies have reported that lncRNA could regulate TF activity through reciprocal interaction [62]. To address this possibility, our web server is equipped to determine whether the co-expression gene set is enriched in targets of specific TFs. Extensive TF-target pairs were first built by annotating two sources of data: 1) computational motif scan of TF binding sites, and 2) experimental TF binding sites as archived by the ENCODE ChIP data. For the latter, we retrieved ENCODE ChIP-seq data and defined the promoter region as a window from -3000 bp to +1000 bp of the transcription start site to establish putative TF occupancy. The output of this type of analysis can be accessed via gene enrichment module.

Analytic module #2: RBP interactome mapping

Second, based on the lncRNAs that have been reported thus far, they have been mostly implicated in several aspects of gene expression, such as RNA stability, miRNA sponging, regulation of transcription factor, epigenetic and chromosome architecture [4, 7, 20, 21, 63]. Interestingly, behind these regulatory actions, molecular interactions are the most crucial determinant in lncRNAs' roles. In this context, lncRNAs are known to associate with various proteins (i.e. RNA-binding proteins and chromatin modifiers). For example, lncRNA *ELFN1-AS1* interacts with hnRNPK to transcriptionally suppress the expression of *CDKN2B,* a tumor suppressor gene [61]. LncRNA *NORAD* acts as sequester of PUM2 to maintain genomic stability [64]. A CRC-associated lncRNA MYU binds hnRNPK and consequently stabilizes CDK6, which is critical for colon cancer cells growth [65]. These findings thus suggest that delineating the lncRNA-interacting protein network may effectively prompt the functional exploration of lncRNA candidates. In our efforts of mapping the protein interactome of lncRNAs, we have extensively curated and integrated two types of public data into reference annotations for the analytic workflow: computational RNA binding protein (RBP) motif scan and experimental RBP databases.

For this purpose, we first collected RBP binding motifs from MEME, which is a motif discovering software, in addition to several RBP motifs from published data [66]. Next, we generated all lncRNAs sequences from GENCODE Release 25 and used

FIMO to scan computationally for the presence of possible RBP binding sites [52]. For the empirical RBP sites, we retrieved the RBP binding sequences from ENCODE eCLIP [67]. To complement the repertoire of RBP included in the analysis, we also integrated protein interaction profile sequencing (PIP-seq) [68]. Although the footprints of protein binding do not readily reveal the identity of the associated factors, PIP-seq data may serve as evidence for molecular interaction.

Given that our exemplary lncRNA *ELFN1-AS1* reportedly mediates its function through interacting with hnRNPK, we next tested whether this attribute could be recapitulated by circlncRNAnet. To interrogate the *ELFN1-AS1*-associated proteins, the "Retrieve lncRNA-binding protein" module can be selected to display *ELFN1-AS1*-associated RNA-binding protein network (Fig. 5A). An RBP is considered a hit (i.e. potential interactor of the given lncRNA/circRNA) if its annotated motifs from at least two database sources are detected in the transcript sequence, and will be labeled with gene symbol and a larger node size. The output of this demo analysis illustrates a number of putative interacting RBPs, one of which is HNRNPK, as reported (Fig. 5A).

Analytic module #3: ceRNA networking

Third, aside from protein interactors, the role of circRNAs/lncRNAs in microRNA (miRNA)-mediated post-transcriptional regulation has emerged. By virtue of the distinct distribution of recurring miRNA target sequences in lncRNA transcripts, certain lncRNAs are known to compete with mRNA transcripts for complementary binding by the cognate miRNAs. This regulatory process, referred to as miRNA sponge or competing endogenous RNAs (ceRNAs) [69], alters the endogenous silencing activity of miRNAs, thereby impacting the expression of targeted mRNAs. Some lncRNAs have even been demonstrated as miRNA sponge in certain oncogenic processes [11, 12]. Thus, to complete this bioinformatics package, we installed in this web server an analytic module for sequence-based delineation of potential lncRNA-miRNA sponge pairs. Given that existing miRNA targeting sites databases annotate target sequences only in 3' UTR, information regarding miRNA:ncRNA complementarity is not readily available. To resolve this issue, we generated a reference database that catalogues putative miRNA binding sites within lncRNAs/circRNAs as computationally predicted by three different miRNA target prediction tools (RNAhybrid, miRanda and TarPmiR) [55, 70, 71]. Analogous to the RBP module, a miRNA target is considered a positive hit if two of the three software tools uncover its existence, and will be denoted as larger node and shown with gene symbol in the network diagram.

For the RNA components of the *ELFN1-AS1* interactomes, circlncRNAnet provides information on the putative miRNA targeting sites within the RNA sequences. To explore, the “miRNA targeting sites network” may be selected to show the corresponding network (Fig. 5B). Analogous to the RBP network, any miRNA target sequences predicted by at least two miRNA targeting site discovering software (miRanda, RNAhybrid and TarPmiR) will be labeled with gene symbols and larger node size in the network (Fig. 5B).

Analytic module #4: Multi-tier regulatory hierarchy

mRNAs harboring the same miRNA binding sites as ncRNAs are likely to be subject to expression alteration in the miRNA sponge scenario – the inverse correlation in expression between miRNA and mRNAs/lncRNAs is expected [69]. Thus, to

substantiate the putative miRNA sponge activity and also to delineate likely downstream mRNA targets, the web server is further designed to construct the ncRNA-miRNA-mRNAs regulatory hierarchy. For this purpose, 3' UTRs with presumptive miRNA targeting as revealed by the aforementioned prediction tools will be cross-referenced with the gene set that shows correlated expression profiles with the candidate ncRNA. As a result, this intersected gene list presumably represent the targets of ncRNA-miRNA axis-mediated regulation, and will be depicted in a two-tier network configuration (Fig. 5B).

Similar network analyses are available for decoding the ncRNA-RBP-mRNA network. To this end, a reference RBP-mRNA database was first established, in which all GENCODE mRNA genes were scanned and annotated for experimental and computational RBP binding using the above approaches. For a particular RBP in the ncRNA interactome that is selected by the user, all ncRNA-co-expressed mRNAs with mutual RBP binding will be assembled based on the RPB-mRNA database. These lines of information will then be integrated and subsequently outputted as the multi-tier molecular network (Fig. 5A).

Benchmarking

circlncRNAnet is constructed on the Nginx 1.6.3 and Shiny 1.0.3 server, which runs on a CentOS 6.2 with two Intel XEON E5-2620 CPU and 200GB RAM. To optimize the CPU utilities for multiple users, we assign two threads for an analysis task. We tested the web service with 20 normal/tumor paired samples, for which the DESeq2 analysis required 130 seconds to produce differentially expressed genes. For calculating co-expressed gene list, circlncRNAnet took 50 seconds for one query gene and 270 seconds for ten query genes.

Conclusions

With the expansion of transcriptome sequencing datasets, focusing on a select set of publicly available, but potentially irrelevant, sequencing data does not sufficiently address users' research needs. This prompted us to build a completely new system with the flexibility of accepting private or public data. To further support efficient analyses and presentation, we have extensively curated public data into reference annotations for the circlncRNAnet workflow. Multi-layer modules and algorithms then provide outputs on expression profiles, co-expression networks & pathways, and molecular interactomes, which are dynamically and interactively displayed according to user-defined criteria. In short, users may apply circlncRNAnet to obtain, in real time, multiple lines of functionally relevant information on the circRNAs/lncRNAs of their interest. The overall workflow takes only a few minutes, as compared to hours of manual efforts of independent database searches and analyses. In summary, circlncRNAnet is the first of its kind in the regulatory RNA research field, providing a "one-stop" resource for in-depth analyses of ncRNA biology. A tutorial with demo datasets is available under "Tutorial", in which the functional network of known lncRNA was illustrated *in silico* as an example.

Availability of supporting source code and requirements

- Project name: circlncRNAnet

- Project home page: http://app.cgu.edu.tw/circlnc/ [72], https://github.com/smw1414/circlncRNAnet [73]
- Operating system(s): Platform independent
- Programming language: PHP, JavaScript, R, R shiny and Shell script
- Other requirements: JavaScript supporting web browser
- License: GPLv3
- Research Resource Identifier: circlncRNAnet, RRID:SCR_015794

Availability of supporting data

The analytic modules and test datasets (from TCGA and ENCODE) are available in the GitHub repository [73]. An archival copy of the modules and test datasets are also available via the *GigaScience* GigaDB repository [74]. For the convenience of prospective users, we also provided on GitHub instructions on running our pipeline in local mode.

Declarations

Abbreviations

ceRNA: competing endogenous RNA; circRNA: circular RNA; CNC: coding–non-coding co-expression; COAD: Colon adenocarcinoma; GSEA: Gene Set Enrichment Analysis; lncRNAs: long non-coding RNA; miRNA: microRNA; mRNAs: messenger RNA; ncRNA: non-coding RNA; PIP-seq: Protein Interaction Profile sequencing; RBP: RNA-binding protein; READ: Rectal adenocarcinoma; TCGA: The Cancer Genome Atlas.

Acknowledgements

We are grateful to members of the BC-MT laboratory for critical reading of the article and important discussions. This work was supported by grants from the Ministry of Science and Technology of Taiwan (MOST104-2321-B-182-007-MY3 to P-JH; MOST106-2320-B-182-035-MY3 to HL; MOST104-2320-B-182-029-MY3 and MOST105-2314-B-182-061-MY4 to BC-MT; MOST103-2632-B-182-001, MOST104-2632-B-182-001, and MOST105-2632-B-182-001), Chang Gung Memorial Hospital (CMRPD1G0321 and CMRPD1G0322 to P-JH; CMRPD1F0571 to HL; CMRPG3D1513 and CMRPG3D1514 to W-ST; CMRPD3E0153, CMRPD1F0442, and BMRP960 to BC-MT), the National Health Research Institute of Taiwan (NHRI-EX105-10321SI), the Ministry of Education of Taiwan, and Biosignature Research Grant CIRPD3B0013 for supporting bioinformatics and computing resources.

Authors' contributions

HL and BCT conceived the original idea of the web server. SW, PH, YC, CL, WT and HL designed and implemented the web server. SW, PJ and YC conducted the benchmarks. CL, CY, WT and BCT tested the system, provided feedback on features and functionality. SW, HL and BCT wrote the manuscript. All authors read and approved the final manuscript.

Competing interests

The authors declare that they have no competing interests.

Tables

Table 1. Comparative functionalities of available web tools of ncRNAs.

Tool name	Interface	Both lncRNAs and circRNAs	Expression pattern	Co-expression: gene network	Co-expression: annotation/ pathway	RBP binding site prediction	miRNA target prediction	Regulatory Network	Ref.
circlncRNAnet	Web server	Yes	Yes	Yes	Yes	Yes	Yes	Yes	This article
NONCODE	Web database		Yes						[25]
LNCipedia	Web database						Yes		[26]
ncFANs	Web server			Yes	Yes				[27]
lncRNAdb	Web database		Yes			Yes	Yes		[28]
LINC	R package			Yes	Yes				[29]
cogena	R package			Yes	Yes				[30]
WGCNA	R package			Yes					[31]
QUBIC	R package			Yes					[32]
circNet	Web database		Yes				Yes	Yes	[33]
CIRCpedia	Web database		Yes						[34]
Circ2Traits	Web database	Yes	Yes		Yes		Yes		[35]
CircInteractome	Web database					Yes	Yes	Yes	[36]
DeepBase V2.0	Web database	Yes	Yes						[24]
starBase V2.0	Web database	Yes			Yes	Yes	Yes	Yes	[37]

Table 2. Analytic and visualization R packages incorporated in circlncRNAnet.

Analytic software	Version	Description	Ref.
circlize	0.4.1	Circos plot	[43]
clusterProfiler	3.2.14	Gene enrichment analysis	[44]
DESeq2	1.14.1	Differential expression analysis	[45]
factoextra	1.0.4	PCA analysis	[46]
ggplot2	2.2.1.9000	Data visualization	[47]
plotly	4.7.1	Interactive data visualization	[48]
visNetwork	2.0.1	Network visualization	[49]
WGCNA	1.51	Correlation calculation	[31]

Table 3. List of databases and analytic tools employed by circlncRNAnet.

Database	Version	Description	Parameters	Ref.
cisBP-RNA and Ray, 2013 (Homo sapiens)	2013	RNA binding protein motifs for FIMO to discover potential RNA binding sites	Downloaded from MEME motif database	[50]
dbNSFP (Homo sapiens)	3.2	Gene annotation	NA	[51]
ENCODE ChIP-Seq (Homo sapiens)	Feb 2017	Experimental transcription factor and protein binding sites	Regions from -3000 ~ 1000 bp of TSS were considered as the promoter. In-house scripts were then used to collect peaks with > 2 score and annotate as binding sites	[41]
ENCODE eCLIP (Homo sapiens)	Mar 2017	Experimental RNA binding protein binding sites	In-house scripts were used to collect all the peaks corresponding to binding sites; binding score for each target gene was represented by the lowest peak score	[41]
FIMO	4.11.2	Computational RNA binding protein binding sites discovering	Default	[52]
GENCODE (Homo sapiens)	Release 25	lncRNA annotation	NA	[23]
LNCipedia (Homo sapiens)	4	High-confidence lncRNA annotation	NA	[26]
miRanda	3.3a	miRNA binding sites detection	-m 10000000 -p 0.05	[53]
MSigDB	v5.2	Computational transcription factor and protein binding sites	The transcription factor targets dataset was used for TF enrichment analysis	[54]
RNAhybrid	2.1.2	miRNA binding sites detection	-sc 140, with cutoff seed similarity>=85% & wobble pair similarity >=85%	[55]
TarPmiR	Mar 2016	miRNA binding sites detection	-p 0.1	[56]

Figure legends

Figure 1. The overall design and the analytic workflow of circlncRNAnet.

Figure 2. Input file formats for circlncRNAnet.
Interface on the web server for data upload (A). Two files are uploaded prior to data analysis: a gene matrix table (B), which is generated by using featureCounts, and a condition file describing the sample status (C).

Figure 3. Schematic showing example outputs of circlncRNAnet analyses of lncRNA-based networks in colorectal cancer.
After dataset upload, the server executes differential expression and expression correlation analyses. The web server allows user to select query genes and correlation criteria (A). For an overview of the sequenced transcriptomes, extent of the coordinated expression (B) and overall distribution of non-coding and coding RNA abundance (C) are displayed as summary graphs. As examples of use, co-expression network analysis of a known lncRNA, ELFN1-AS1, and novel lncRNA, XXbac-B476C20.9, was performed using circlncRNAnet. (D) Scatter plot showing the extent of expression correlation between ELFNA-AS1 and one target, MYC. (E) Histogram displaying the distributions of the Pearson correlation coefficients of all ncRNA-mRNA pairs (Obs) and of a randomized correlation test (Rand).

Figure 4. Additional examples of circlncRNAnet output of lncRNA-based networks in colorectal cancer.
In addition to analyses shown in Figure 3, more options for network interrogation of ncRNA-based regulation can be accessed on the webpage (middle). For instance, heatmap representation of the genes co-expressed with ELFN1-AS1 (Pearson's $|r| > 0.5$) can be outputted (upper left). Pathway analysis of the co-expressed genes on the basis of MSigdb Hallmark pathways (bottom left), and its network depiction of top 3 enriched pathways and the corresponding co-expressed components (bottom right). Circos plot can also be used to illustrate the genome-wide distribution of the top 100 co-expressed genes relative to the location of *XXbac-B476C20.9* (upper right).

Figure 5. Examples of lncRNA-associated molecular components uncovered by circlncRNAnet.
circlncRNAnet may be used to extensively profile the molecular interactome of candidate circRNAs/lncRNAs based on the compiled databases, with ELFN1-AS1 shown as an example in this figure. (A) For the RBP components, the interactome will be outputted in both the table format (top) and network configuration (bottom). (B) Similarly, for the putative miRNA sponge network, predicted ELFN1-AS1-targeting miRNAs are shown in table (top) as well as network (bottom) formats. The web server is also designed to construct the ncRNA-RBP-mRNAs or ncRNA-miRNA-mRNAs regulatory hierarchy. circlncRNAnet delineates co-expressed mRNA genes with mutually shared RBP binding or miRNA targeting sites. Consequently, an intersected gene list is compiled (top) and may be depicted in a two-tier network configuration (bottom).

References

1. Ponting CP, Oliver PL and Reik W. Evolution and functions of long noncoding RNAs. *Cell*. 2009;136 4:629-41. doi:10.1016/j.cell.2009.02.006.
2. Liu J, Liu T, Wang X and He A. Circles reshaping the RNA world: from waste to treasure. *Mol Cancer*. 2017;16 1:58. doi:10.1186/s12943-017-0630-y.
3. Quinn JJ and Chang HY. Unique features of long non-coding RNA biogenesis and function. *Nat Rev Genet*. 2016;17 1:47-62. doi:10.1038/nrg.2015.10.
4. Wang KC and Chang HY. Molecular mechanisms of long noncoding RNAs. *Mol Cell*. 2011;43 6:904-14. doi:10.1016/j.molcel.2011.08.018.
5. Jeck WR and Sharpless NE. Detecting and characterizing circular RNAs. *Nat Biotechnol*. 2014;32 5:453-61. doi:10.1038/nbt.2890.
6. Petkovic S and Muller S. RNA circularization strategies in vivo and in vitro. *Nucleic Acids Res*. 2015;43 4:2454-65. doi:10.1093/nar/gkv045.
7. Rinn JL and Chang HY. Genome regulation by long noncoding RNAs. *Annu Rev Biochem*. 2012;81:145-66. doi:10.1146/annurev-biochem-051410-092902.
8. Wang X, Arai S, Song X, Reichart D, Du K, Pascual G, et al. Induced ncRNAs allosterically modify RNA-binding proteins in cis to inhibit transcription. *Nature*. 2008;454 7200:126-30. doi:10.1038/nature06992.
9. Guttman M and Rinn JL. Modular regulatory principles of large non-coding RNAs. *Nature*. 2012;482 7385:339-46. doi:10.1038/nature10887.
10. Rinn JL, Kertesz M, Wang JK, Squazzo SL, Xu X, Brugmann SA, et al. Functional demarcation of active and silent chromatin domains in human HOX loci by noncoding RNAs. *Cell*. 2007;129 7:1311-23. doi:10.1016/j.cell.2007.05.022.
11. Shao Y, Ye M, Li Q, Sun W, Ye G, Zhang X, et al. LncRNA-RMRP promotes carcinogenesis by acting as a miR-206 sponge and is used as a novel biomarker for gastric cancer. *Oncotarget*. 2016;7 25:37812-24. doi:10.18632/oncotarget.9336.
12. Du Z, Sun T, Hacisuleyman E, Fei T, Wang X, Brown M, et al. Integrative analyses reveal a long noncoding RNA-mediated sponge regulatory network in prostate cancer. *Nat Commun*. 2016;7:10982. doi:10.1038/ncomms10982.
13. Boeckel JN, Jae N, Heumuller AW, Chen W, Boon RA, Stellos K, et al. Identification and Characterization of Hypoxia-Regulated Endothelial Circular RNA. *Circulation research*. 2015;117 10:884-90. doi:10.1161/CIRCRESAHA.115.306319.
14. Militello G, Weirick T, John D, Doring C, Dimmeler S and Uchida S. Screening and validation of lncRNAs and circRNAs as miRNA sponges. *Briefings in bioinformatics*. 2016; doi:10.1093/bib/bbw053.
15. Chen G, Wang Z, Wang D, Qiu C, Liu M, Chen X, et al. LncRNADisease: a database for long-non-coding RNA-associated diseases. *Nucleic Acids Res*. 2013;41 Database issue:D983-6. doi:10.1093/nar/gks1099.
16. Huarte M. RNA. A lncRNA links genomic variation with celiac disease. *Science*. 2016;352 6281:43-4. doi:10.1126/science.aaf6015.

17. Li P, Chen S, Chen H, Mo X, Li T, Shao Y, et al. Using circular RNA as a novel type of biomarker in the screening of gastric cancer. *Clin Chim Acta*. 2015;444:132-6. doi:10.1016/j.cca.2015.02.018.
18. Qian Y, Lu Y, Rui C, Qian Y, Cai M and Jia R. Potential Significance of Circular RNA in Human Placental Tissue for Patients with Preeclampsia. *Cell Physiol Biochem*. 2016;39 4:1380-90. doi:10.1159/000447842.
19. Yang G, Lu X and Yuan L. LncRNA: a link between RNA and cancer. *Biochim Biophys Acta*. 2014;1839 11:1097-109. doi:10.1016/j.bbagrm.2014.08.012.
20. Xie X, Tang B, Xiao YF, Xie R, Li BS, Dong H, et al. Long non-coding RNAs in colorectal cancer. *Oncotarget*. 2016;7 5:5226-39. doi:10.18632/oncotarget.6446.
21. Han D, Wang M, Ma N, Xu Y, Jiang Y and Gao X. Long noncoding RNAs: novel players in colorectal cancer. *Cancer Lett*. 2015;361 1:13-21. doi:10.1016/j.canlet.2015.03.002.
22. Park NJ, Zhou H, Elashoff D, Henson BS, Kastratovic DA, Abemayor E, et al. Salivary microRNA: discovery, characterization, and clinical utility for oral cancer detection. *Clin Cancer Res*. 2009;15 17:5473-7. doi:10.1158/1078-0432.CCR-09-0736.
23. Harrow J, Frankish A, Gonzalez JM, Tapanari E, Diekhans M, Kokocinski F, et al. GENCODE: the reference human genome annotation for The ENCODE Project. *Genome Res*. 2012;22 9:1760-74. doi:10.1101/gr.135350.111.
24. Zheng LL, Li JH, Wu J, Sun WJ, Liu S, Wang ZL, et al. deepBase v2.0: identification, expression, evolution and function of small RNAs, LncRNAs and circular RNAs from deep-sequencing data. *Nucleic Acids Res*. 2016;44 D1:D196-202. doi:10.1093/nar/gkv1273.
25. Zhao Y, Li H, Fang S, Kang Y, Wu W, Hao Y, et al. NONCODE 2016: an informative and valuable data source of long non-coding RNAs. *Nucleic Acids Res*. 2016;44 D1:D203-8. doi:10.1093/nar/gkv1252.
26. Volders PJ, Verheggen K, Menschaert G, Vandepoele K, Martens L, Vandesompele J, et al. An update on LNCipedia: a database for annotated human lncRNA sequences. *Nucleic Acids Res*. 2015;43 8:4363-4. doi:10.1093/nar/gkv295.
27. Liao Q, Xiao H, Bu D, Xie C, Miao R, Luo H, et al. ncFANs: a web server for functional annotation of long non-coding RNAs. *Nucleic Acids Res*. 2011;39 Web Server issue:W118-24. doi:10.1093/nar/gkr432.
28. Quek XC, Thomson DW, Maag JL, Bartonicek N, Signal B, Clark MB, et al. lncRNAdb v2.0: expanding the reference database for functional long noncoding RNAs. *Nucleic Acids Res*. 2015;43 Database issue:D168-73. doi:10.1093/nar/gku988.
29. Goepferich M and Herrmann C. LINC: co-expression of lincRNAs and protein-coding genes. 2017.
30. Jia Z and Barnes M. cogena: co-expressed gene-set enrichment analysis. 2017.
31. Langfelder P and Horvath S. WGCNA: an R package for weighted correlation network analysis. *BMC Bioinformatics*. 2008;9:559. doi:10.1186/1471-2105-9-559.

32. Zhang Y, Xie J, Yang J, Fennell A, Zhang C and Ma Q. QUBIC: a bioconductor package for qualitative biclustering analysis of gene co-expression data. *Bioinformatics*. 2016; doi:10.1093/bioinformatics/btw635.
33. Liu YC, Li JR, Sun CH, Andrews E, Chao RF, Lin FM, et al. CircNet: a database of circular RNAs derived from transcriptome sequencing data. *Nucleic Acids Res*. 2016;44 D1:D209-15. doi:10.1093/nar/gkv940.
34. Zhang XO, Dong R, Zhang Y, Zhang JL, Luo Z, Zhang J, et al. Diverse alternative back-splicing and alternative splicing landscape of circular RNAs. *Genome Res*. 2016;26 9:1277-87. doi:10.1101/gr.202895.115.
35. Ghosal S, Das S, Sen R, Basak P and Chakrabarti J. Circ2Traits: a comprehensive database for circular RNA potentially associated with disease and traits. *Front Genet*. 2013;4:283. doi:10.3389/fgene.2013.00283.
36. Dudekula DB, Panda AC, Grammatikakis I, De S, Abdelmohsen K and Gorospe M. CircInteractome: A web tool for exploring circular RNAs and their interacting proteins and microRNAs. *RNA Biol*. 2016;13 1:34-42. doi:10.1080/15476286.2015.1128065.
37. Li JH, Liu S, Zhou H, Qu LH and Yang JH. starBase v2.0: decoding miRNA-ceRNA, miRNA-ncRNA and protein-RNA interaction networks from large-scale CLIP-Seq data. *Nucleic Acids Res*. 2014;42 Database issue:D92-7. doi:10.1093/nar/gkt1248.
38. Liao Y, Smyth GK and Shi W. featureCounts: an efficient general purpose program for assigning sequence reads to genomic features. *Bioinformatics*. 2014;30 7:923-30. doi:10.1093/bioinformatics/btt656.
39. Szabo L, Morey R, Palpant NJ, Wang PL, Afari N, Jiang C, et al. Statistically based splicing detection reveals neural enrichment and tissue-specific induction of circular RNA during human fetal development. *Genome Biol*. 2015;16:126. doi:10.1186/s13059-015-0690-5.
40. Cancer Genome Atlas N. Comprehensive molecular characterization of human colon and rectal cancer. *Nature*. 2012;487 7407:330-7. doi:10.1038/nature11252.
41. Consortium EP. An integrated encyclopedia of DNA elements in the human genome. *Nature*. 2012;489 7414:57-74. doi:10.1038/nature11247.
42. Glazar P, Papavasileiou P and Rajewsky N. circBase: a database for circular RNAs. *RNA*. 2014;20 11:1666-70. doi:10.1261/rna.043687.113.
43. Gu Z, Gu L, Eils R, Schlesner M and Brors B. circlize Implements and enhances circular visualization in R. *Bioinformatics*. 2014;30 19:2811-2. doi:10.1093/bioinformatics/btu393.
44. Yu G, Wang LG, Han Y and He QY. clusterProfiler: an R package for comparing biological themes among gene clusters. *OMICS*. 2012;16 5:284-7. doi:10.1089/omi.2011.0118.
45. Love MI, Huber W and Anders S. Moderated estimation of fold change and dispersion for RNA-seq data with DESeq2. *Genome Biol*. 2014;15 12:550. doi:10.1186/s13059-014-0550-8.
46. Mundt AKF. factoextra: Extract and Visualize the Results of Multivariate Data Analyses. 2017.
47. Wickham H. ggplot2: Elegant Graphics for Data Analysis. Springer-Verlag New York; 2009.

48. Sievert C, Parmer C, Hocking T, Chamberlain S, Ram K, Corvellec M, et al. plotly: Create Interactive Web Graphics via 'plotly.js'. 2017.
49. B.V. A, Thieurmel B and Robert T. visNetwork: Network Visualization using 'vis.js' Library. 2017.
50. Ray D, Kazan H, Cook KB, Weirauch MT, Najafabadi HS, Li X, et al. A compendium of RNA-binding motifs for decoding gene regulation. *Nature*. 2013;499 7457:172-7. doi:10.1038/nature12311.
51. Liu X, Jian X and Boerwinkle E. dbNSFP: a lightweight database of human nonsynonymous SNPs and their functional predictions. *Hum Mutat*. 2011;32 8:894-9. doi:10.1002/humu.21517.
52. Grant CE, Bailey TL and Noble WS. FIMO: scanning for occurrences of a given motif. *Bioinformatics*. 2011;27 7:1017-8. doi:10.1093/bioinformatics/btr064.
53. John B, Enright AJ, Aravin A, Tuschl T, Sander C and Marks DS. Human MicroRNA targets. *PLoS Biol*. 2004;2 11:e363. doi:10.1371/journal.pbio.0020363.
54. Subramanian A, Tamayo P, Mootha VK, Mukherjee S, Ebert BL, Gillette MA, et al. Gene set enrichment analysis: a knowledge-based approach for interpreting genome-wide expression profiles. *Proc Natl Acad Sci U S A*. 2005;102 43:15545-50. doi:10.1073/pnas.0506580102.
55. Kruger J and Rehmsmeier M. RNAhybrid: microRNA target prediction easy, fast and flexible. *Nucleic Acids Res*. 2006;34 Web Server issue:W451-4. doi:10.1093/nar/gkl243.
56. Ding J, Li X and Hu H. TarPmiR: a new approach for microRNA target site prediction. *Bioinformatics*. 2016;32 18:2768-75. doi:10.1093/bioinformatics/btw318.
57. De Smet R and Marchal K. Advantages and limitations of current network inference methods. *Nat Rev Microbiol*. 2010;8 10:717-29. doi:10.1038/nrmicro2419.
58. Wolfe CJ, Kohane IS and Butte AJ. Systematic survey reveals general applicability of "guilt-by-association" within gene coexpression networks. *BMC Bioinformatics*. 2005;6:227. doi:10.1186/1471-2105-6-227.
59. Ricano-Ponce I, Zhernakova DV, Deelen P, Luo O, Li X, Isaacs A, et al. Refined mapping of autoimmune disease associated genetic variants with gene expression suggests an important role for non-coding RNAs. *J Autoimmun*. 2016;68:62-74. doi:10.1016/j.jaut.2016.01.002.
60. D'Haene E, Jacobs EZ, Volders PJ, De Meyer T, Menten B and Vergult S. Identification of long non-coding RNAs involved in neuronal development and intellectual disability. *Sci Rep*. 2016;6:28396. doi:10.1038/srep28396.
61. Kim T, Jeon YJ, Cui R, Lee JH, Peng Y, Kim SH, et al. Role of MYC-regulated long noncoding RNAs in cell cycle regulation and tumorigenesis. *J Natl Cancer Inst*. 2015;107 4 doi:10.1093/jnci/dju505.
62. Fu M, Huang G, Zhang Z, Liu J, Zhang Z, Huang Z, et al. Expression profile of long noncoding RNAs in cartilage from knee osteoarthritis patients. *Osteoarthritis Cartilage*. 2015;23 3:423-32. doi:10.1016/j.joca.2014.12.001.
63. Chen X, Liu B, Yang R, Guo Y, Li F, Wang L, et al. Integrated analysis of long non-coding RNAs in human colorectal cancer. *Oncotarget*. 2016;7 17:23897-908. doi:10.18632/oncotarget.8192.

64. Lee S, Kopp F, Chang TC, Sataluri A, Chen B, Sivakumar S, et al. Noncoding RNA NORAD Regulates Genomic Stability by Sequestering PUMILIO Proteins. *Cell*. 2016;164 1-2:69-80. doi:10.1016/j.cell.2015.12.017.
65. Kawasaki Y, Komiya M, Matsumura K, Negishi L, Suda S, Okuno M, et al. MYU, a Target lncRNA for Wnt/c-Myc Signaling, Mediates Induction of CDK6 to Promote Cell Cycle Progression. *Cell Rep*. 2016;16 10:2554-64. doi:10.1016/j.celrep.2016.08.015.
66. Bailey TL, Boden M, Buske FA, Frith M, Grant CE, Clementi L, et al. MEME SUITE: tools for motif discovery and searching. *Nucleic Acids Res*. 2009;37 Web Server issue:W202-8. doi:10.1093/nar/gkp335.
67. Hong EL, Sloan CA, Chan ET, Davidson JM, Malladi VS, Strattan JS, et al. Principles of metadata organization at the ENCODE data coordination center. *Database (Oxford)*. 2016;2016 doi:10.1093/database/baw001.
68. Silverman IM, Li F, Alexander A, Goff L, Trapnell C, Rinn JL, et al. RNase-mediated protein footprint sequencing reveals protein-binding sites throughout the human transcriptome. *Genome Biol*. 2014;15 1:R3. doi:10.1186/gb-2014-15-1-r3.
69. Thomson DW and Dinger ME. Endogenous microRNA sponges: evidence and controversy. *Nat Rev Genet*. 2016;17 5:272-83. doi:10.1038/nrg.2016.20.
70. Lewis BP, Burge CB and Bartel DP. Conserved seed pairing, often flanked by adenosines, indicates that thousands of human genes are microRNA targets. *Cell*. 2005;120 1:15-20. doi:10.1016/j.cell.2004.12.035.
71. Enright AJ, John B, Gaul U, Tuschl T, Sander C and Marks DS. MicroRNA targets in Drosophila. *Genome Biol*. 2003;5 1:R1. doi:10.1186/gb-2003-5-1-r1.
72. Wu S-M, Liu H, Huang P-J, Chang IY-F, Lee C-C, Yang C-Y, et al.: circlncRNAnet: An integrated web-based resource for mapping functional networks of long or circular forms of non-coding RNAs. http://http://app.cgu.edu.tw/circlnc/ (2017). Accessed November 2017.
73. Wu S-M, Liu H, Huang P-J, Chang IY-F, Lee C-C, Yang C-Y, et al.: circlncRNAnet GitHub repository. https://github.com/smw1414/circlncRNAnet (2017). Accessed November 2017.
74. Wu S-M, Liu H, Huang P-J, Chang IY-F, Lee C-C, Yang C-Y, et al. Supporting data for "circlncRNAnet: An integrated web-based resource for mapping functional networks of long or circular forms of non-coding RNAs". *GigaScience Database*. 2017. http://dx.doi.org/10.5524/100378.

Click here to download Figure fig1.pdf

Figure 1

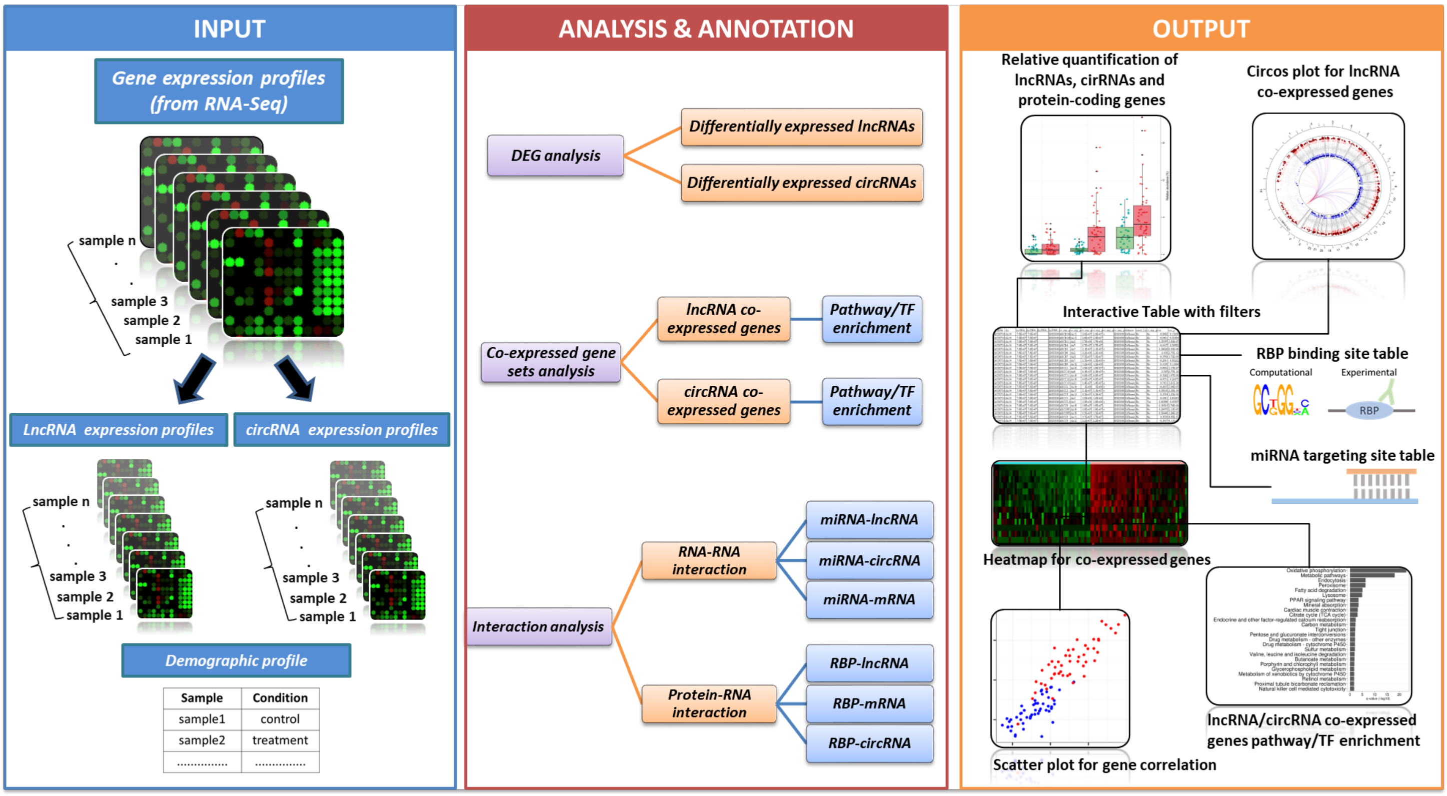

Figure 2 [Click here to download Figure fig2.pdf]

Figure 2

A

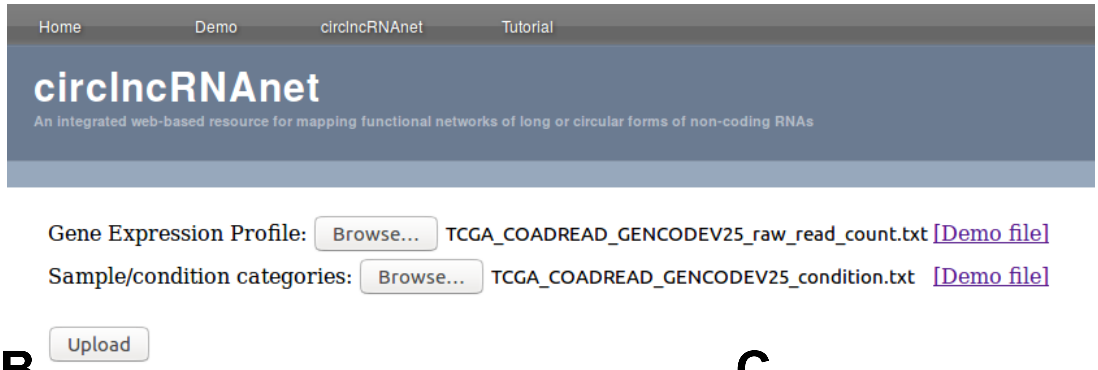

B

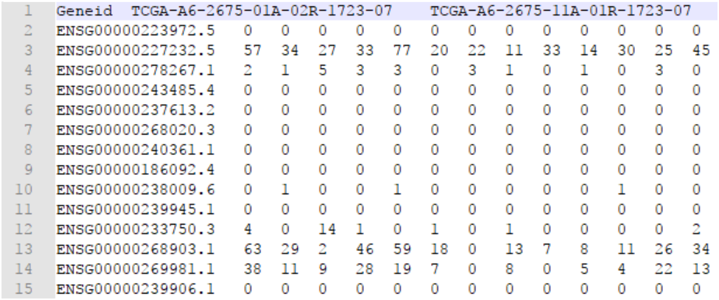

1	Geneid	TCGA-A6-2675-01A-02R-1723-07	TCGA-A6-2675-11A-01R-1723-07											
2	ENSG00000223972.5	0	0	0	0	0	0	0	0	0	0	0	0	0
3	ENSG00000227232.5	57	34	27	33	77	20	22	11	33	14	30	25	45
4	ENSG00000278267.1	2	1	5	3	3	0	3	1	0	1	0	3	0
5	ENSG00000243485.4	0	0	0	0	0	0	0	0	0	0	0	0	0
6	ENSG00000237613.2	0	0	0	0	0	0	0	0	0	0	0	0	0
7	ENSG00000268020.3	0	0	0	0	0	0	0	0	0	0	0	0	0
8	ENSG00000240361.1	0	0	0	0	0	0	0	0	0	0	0	0	0
9	ENSG00000186092.4	0	0	0	0	0	0	0	0	0	0	0	0	0
10	ENSG00000238009.6	0	1	0	0	1	0	0	0	0	0	1	0	0
11	ENSG00000239945.1	0	0	0	0	0	0	0	0	0	0	0	0	0
12	ENSG00000233750.3	4	0	14	1	0	1	0	1	0	0	0	0	2
13	ENSG00000268903.1	63	29	2	46	59	18	0	13	7	8	11	26	34
14	ENSG00000269981.1	38	11	9	28	19	7	0	8	0	5	4	22	13
15	ENSG00000239906.1	0	0	0	0	0	0	0	0	0	0	0	0	0

C

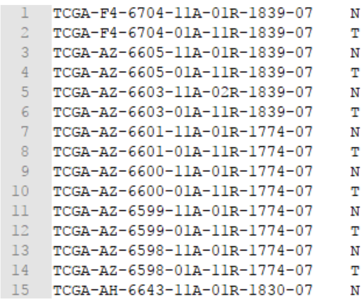

1	TCGA-F4-6704-11A-01R-1839-07	N
2	TCGA-F4-6704-01A-11R-1839-07	T
3	TCGA-AZ-6605-11A-01R-1839-07	N
4	TCGA-AZ-6605-01A-11R-1839-07	T
5	TCGA-AZ-6603-11A-02R-1839-07	N
6	TCGA-AZ-6603-01A-11R-1839-07	T
7	TCGA-AZ-6601-11A-01R-1774-07	N
8	TCGA-AZ-6601-01A-11R-1774-07	T
9	TCGA-AZ-6600-11A-01R-1774-07	N
10	TCGA-AZ-6600-01A-11R-1774-07	T
11	TCGA-AZ-6599-11A-01R-1774-07	N
12	TCGA-AZ-6599-01A-11R-1774-07	T
13	TCGA-AZ-6598-11A-01R-1774-07	N
14	TCGA-AZ-6598-01A-11R-1774-07	T
15	TCGA-AH-6643-11A-01R-1830-07	N

Figure 3

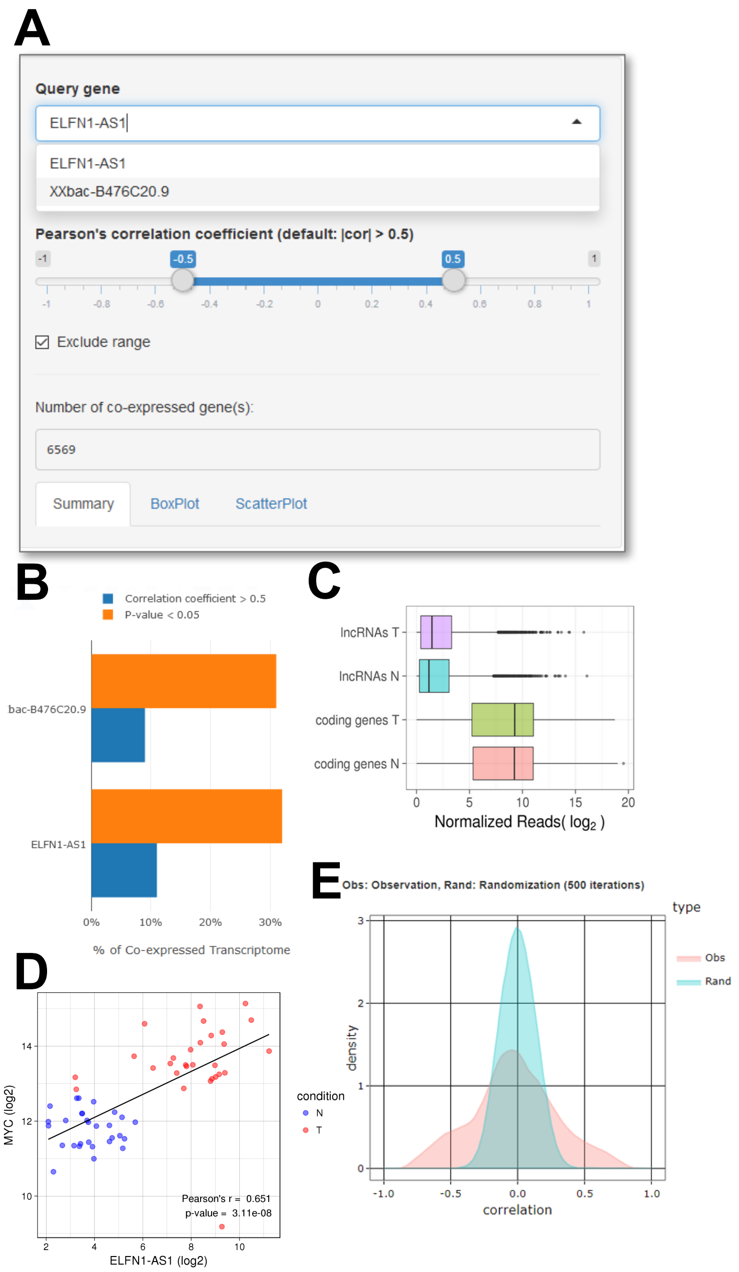
A
Query gene
ELFN1-AS1
ELFN1-AS1
XXbac-B476C20.9
Pearson's correlation coefficient (default: |cor| > 0.5)
-1
-0.5
0.5
1
Exclude range
Number of co-expressed gene(s):
6569
Summary
BoxPlot
ScatterPlot
B
Correlation coefficient > 0.5
P-value < 0.05
bac-B476C20.9
ELFN1-AS1
0%
10%
20%
30%
% of Co-expressed Transcriptome
C
lncRNAs T
lncRNAs N
coding genes T
coding genes N
0
5
10
15
20
Normalized Reads(log2)
D
MYC (log2)
ELFN1-AS1 (log2)
Pearson's r = 0.651
p-value = 3.11e-08
condition
N
T
E
Obs: Observation, Rand: Randomization (500 iterations)
type
Obs
Rand
density
correlation

Click here to download Figure fig4.pdf

Figure 4

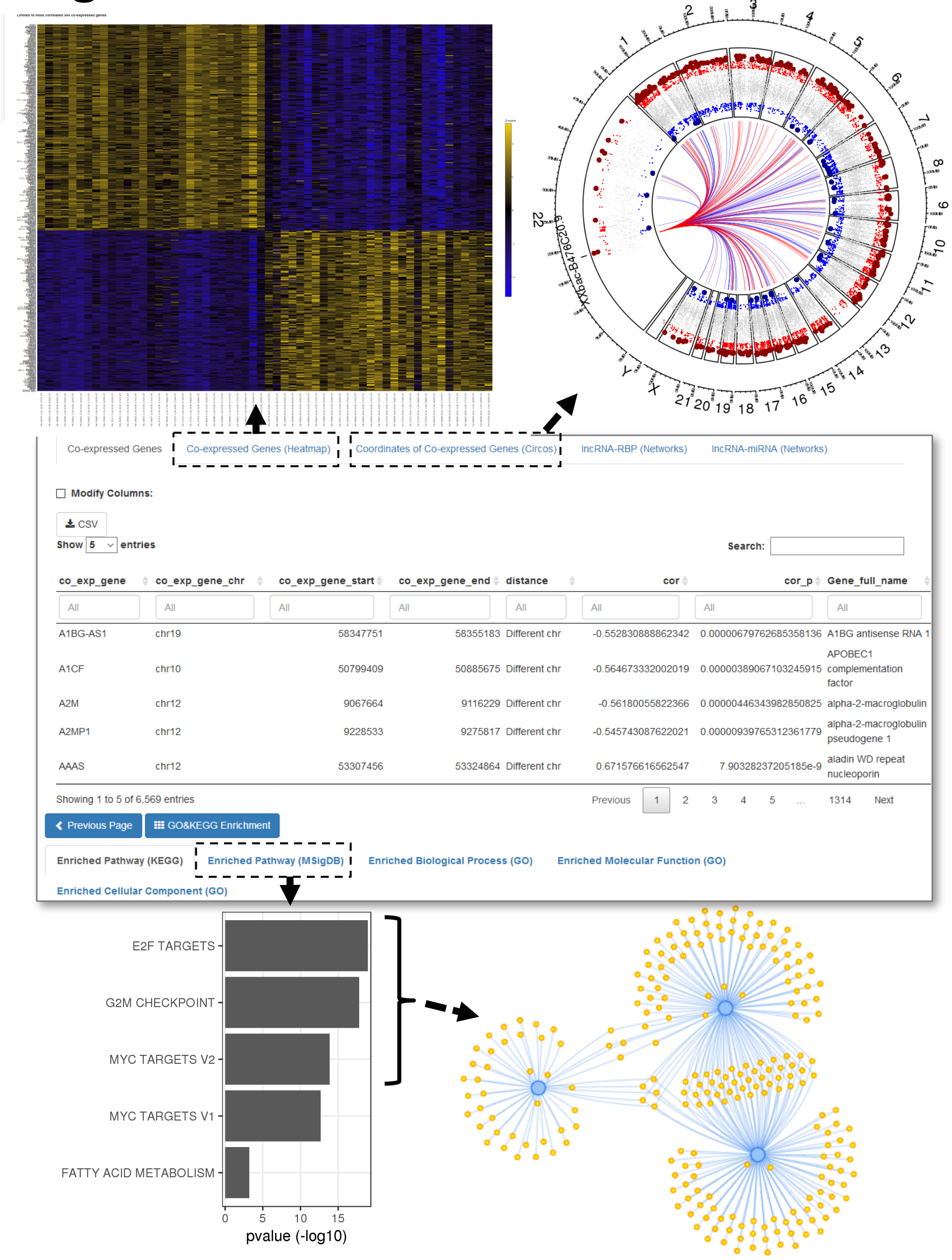

Figure 5 Click here to download Figure fig5.pdf

Figure 5

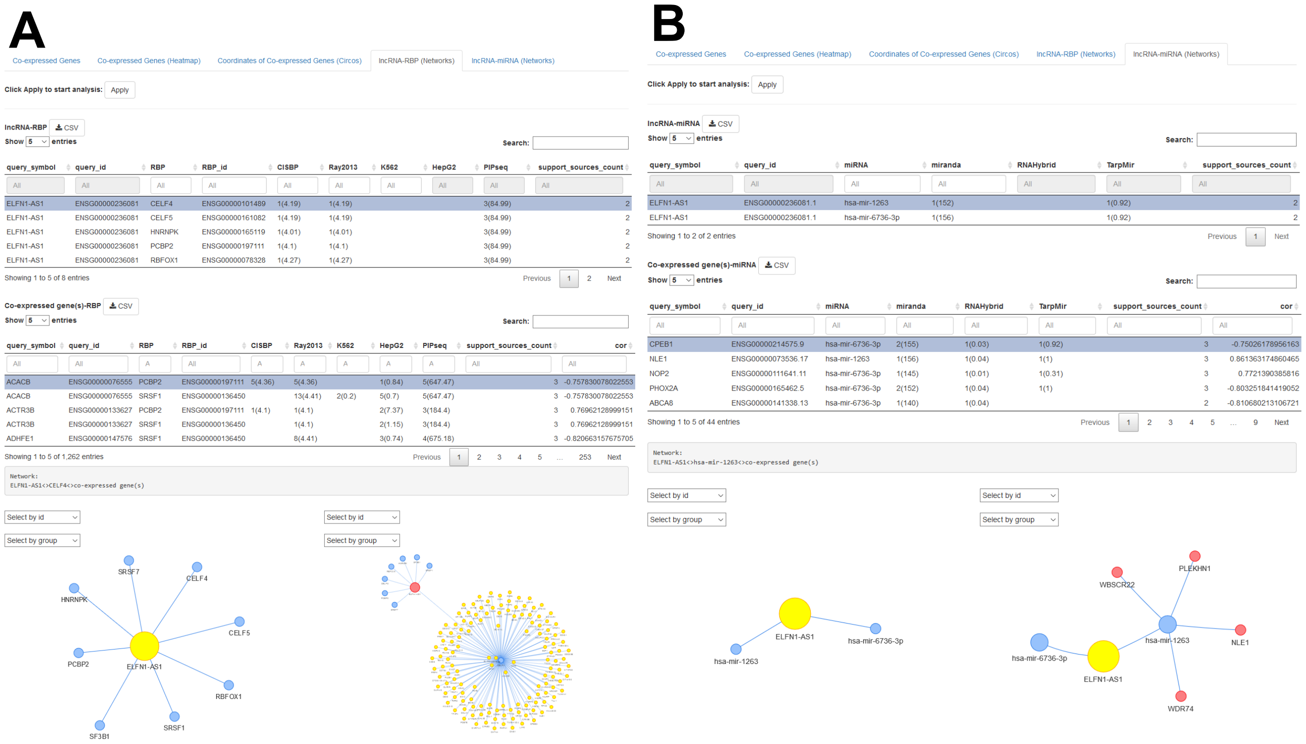

Supplement: GIGA-D-17-00121_Revision-1.pdf [file gix118_giga-d-17-00121_revision-1.pdf]
