# Supplementary material for: circlncRNAnet: an integrated web-based resource for mapping functional networks of long or circular forms of noncoding RNAs
Source: Gigascience. 2017 Nov 29;7(1):gix118. doi: 10.1093/gigascience/gix118 (PMC5765557; doi:10.1093/gigascience/gix118)
Supplement: Response-to-Reviewer-Comments_Original-Submission.pdf [file gix118_response-to-reviewer-comments_original-submission.pdf]

Author's Response To Reviewer Comments

Point-by-point response to referees comments (MS ID# GIGA-D-17-00121_R1)
(>: our responses)
(Modifications and the inclusion of new data have been marked in red in the revised manuscript and summarized in List of amendments to manuscript)

Reviewer #1: Overall the tool implements a wide variety of functionality and appears to function as described. I believe this will be a useful to biologists studying lncRNAs. There are a few areas I believe should be addressed before this paper is ready for publication.

1) Quite a large number of sentences seem to be missing citations.
Ex:
"Both lncRNAs and circRNAs have been found to harbor microRNA response elements (MREs) and potentially act as "miRNA sponges" that sequester these endogenous small RNAs"
I guess you are intending for the citations in the following sentence to cover this sentence as well?

>Thank you for pointing this out. The citations in the following sentence were indeed intended for this sentence. We have moved the citations to the more appropriate location (p. 3, lines 31-32). We also provided more references according to reviewer’s comments below (points #2 and #3).

1) At the moment I believe the writing and formatting is the biggest issue with this paper. These make the article difficult to follow in some sections.

>We appreciate the valuable suggestions on writing. All the suggestions have been addressed accordingly. To improve the overall quality of writing, we have also thoroughly reviewed the text and made all the necessary changes.

1a) The article is verbose, making it difficult to follow.
Ex:
"At the mechanistic level, regardless of the forms, these non-coding RNA transcripts have been known to impact the expression of messenger RNAs (mRNA) via epigenetic and post-transcriptional regulation."

This sentence could be shortened to something like this.
"Non-coding RNAs are known to affect messenger RNA (mRNA) expression via epigenetic and post-transcriptional regulation"

>The suggested modification is now incorporated in the Abstract (page 2, line 5).

1b) The word usage is a bit strange in some places.
Ex:
"To circumvent this problem, we have implemented an integrative bioinformatics approach to approach to examine in silico the cellular roles of lncRNAs by mapping their functional

networks."

Solve is more appropriate instead of circumvent. Circumvent sounds like you are finding a way around the problem without fixing it. Note this sentence is also verbose. I think reducing the verbosity and complexity of the language in the paper will help solve word usage problems.

>Thank you for the suggestion. We have made the corresponding adjustment (page 4, line 9). In addition, we have modified this sentence to improve clarity: “To solve this problem, we have implemented an integrative bioinformatics approach to examine in silico the functional networks of ncRNAs.”

1c) Use more nouns and fewer pronouns.
>We have addressed this issue by making the following changes:
“It” is changed to “The web server” (page 2, line 17).
“They” is changed to “lncRNAs” (page 3, line 14).

2) Many researchers now consider circRNAs to be lncRNAs. Maybe add something like "… and in fact circRNAs are considered to be a class of lncRNAs by many researchers.. (1)" to "Despite their differences in structure and biosynthesis steps, lncRNAs and circRNAs are much more common in terms of their roles and mechanism in gene regulation"
1. Quinn, Jeffrey J., and Howard Y. Chang. "Unique features of long non-coding RNA biogenesis and function." Nature Reviews Genetics 17.1 (2016): 47-62.

>Thank you for the insightful comments and important references (both points #2 and #3). There are indeed certain similarity and distinctions between these two types of ncRNAs. We have now revised the descriptions accordingly. The above publication is cited as new reference #3 in page 3, line 21

3) The evidence lncRNAs acting as miRNA sponges is much stronger than the evidence for circRNAs acting as miRNA sponges. I recommend adding a sentence to the effect of "Although the evidence for lncRNA miRNA sponges is much stronger than circRNA sponges (1). " after "Both lncRNAs and circRNAs have been found to harbor microRNA response elements (MREs) and potentially act as "miRNA sponges" that sequester these endogenous small RNAs"

1. Militello, Giuseppe, et al. "Screening and validation of lncRNAs and circRNAs as miRNA sponges." Briefings in Bioinformatics (2016): bbw053.
2. Boeckel, Jes-Niels, et al. "Identification and characterization of hypoxia-regulated endothelial circular RNA." Circulation research (2015): CIRCRESAHA-115.

>We agree with the reviewer that, due to the unique structure and abundance, lncRNAs may confer a much stronger effect on miRNA sponging. These two references are added as: “… although the evidence for lncRNA miRNA sponges is much stronger than circRNA sponges [13, 14].” (page 3, lines 32-33).

4) I recommend increasing the quality of the github repository by adding comments to your code and enough documentation that a user could set up their own instance of your server.

>Thank you for the kind reminder. We have now included additional information on how the modules were installed and connected. We also provided on GitHub instructions on running our pipeline in local mode.

Reviewer #2: The pipeline and associated website proposed by the authors is of interest for researchers wanting to better characterize one or several non-coding RNAs.

[My first major criticism is that the the website was not accessible on July 29th and August 1st which prevented me to assess the proposed tool. / Editors' note, update 13/8: The reviewer checked again after sending his report, and could meanwhile assess the homepage.]

>We apologize for the temporary shutdown of the web server. There was an unexpected power outage at the building where the server as housed.

The manuscript contains very little detail on how the analyses are performed, I had to check the different scripts available on the GitHub repository was able to find a github repository to understand the analyses performed (differential expression, co-expression analysis). Additionally there is no list of the packages called by the program, nor does the manuscript highlight the species and references supported. Furthermore there is no information regarding the parameters used to run the different programs (miRanda, RNAHybrid ...). The authors need to significantly develop this, it is nice to have examples of the different analyses the pipeline enables but it is of utmost importance that the reader gains a good grasp of the tools and analyses performed by the pipeline and the parameters used.

>We thank the reviewer for this important reminder. These lines of information were available at the time of submission but omitted from the text for space conservation. As suggested by the reviewer, these details on the analyses are now incorporated in the manuscript: differential expression (DESeq2), page 5, lines 3; co-expression analysis (WGCNA), page 5, lines 20-22. Furthermore, we listed in two new tables the software tools (Table2) and databases (Table 3) called by our server. In Table 3 with software tools, we also provided the parameters used to run these programs. We incorporated this information in the “Output summary” section of the Results (page 4, lines 36-37).

Minor comments the authors mention that the users can define the gene list after differential expression using the fold changes and p-values. Are they using the p-value or the adjust p-value? From the scripts available on github (deseq2_annotation.r) it appears that the column used is the uncorrected p-value which implies a list of differentially expressed genes heavily contaminated by false positives.

>Thank you for this reminder. “Adjusted p-values” (p-adjusted or padj on the web site) are now used as the criterion for distinguishing differential expression.

It would be very useful to the user if the authors could guide them in the choice of the Pearson's coefficient of correlation, such as using a randomization approach on the submitted sets to identify the thresholds.

>Selection of appropriate threshold for correlation is indeed important to subsequent network analyses. Randomization approach for threshold selection is now incorporated at this step of data analysis (500 iterations of randomized Pearson correlations between target ncRNA and 5,000 randomly selected mRNAs). As a guide to the users, the server will display a composite histogram showing the overall distribution of correlation coefficients calculated for all the ncRNA-mRNAs pairs, superimposed with the results from randomized correlation tests. This new function is now described as Figure 3E on page 5, lines 25-31.

List of amendments to manuscript (MS ID# GIGA-D-17-00121_R1)
Referees' comments have been addressed in detail in the "Point-by-point response to referees comments". Modifications and the inclusion of new data have been marked in red in the revised manuscript and summarized by the following points:

Abstract:
P2. Minor changes in wording were made in response to reviewer's suggestions (lines 5 and 17).

Introduction:
P3. Two new sentences and corresponding references (#3, #13 and #14) were incorporated in the second paragraph (lines 20-21 and 32-33).

Results and Methods:
P4. Two new tables (Tables 2 and 3) and corresponding descriptions were added in the "Output summary" section (lines 36-37).
P4-5. Descriptions for several analyses, such as differential expression (p. 5, line 3) expression correlation (p. 5, lines 20-22), as well as randomized correlation test (p. 5, lines 25-31), were added in the "Analytic module #1: coding–non-coding co-expression (CNC) network profiling" section.

Availability of supporting data:
P9. For the convenience of prospective users, we also provided on GitHub instructions on running our pipeline in local mode. This statement is added in lines 10-12.

GitHub repository:
We provided GitHub additional details on how the modules are executed and also instructions on running our pipeline in local mode.

Figures and Legends:
Figure 3E: An exemplary histogram of randomized test results, together with the corresponding legends, were incorporated.
