# Supplementary material for: circlncRNAnet: an integrated web-based resource for mapping functional networks of long or circular forms of noncoding RNAs
Source: Gigascience. 2017 Nov 29;7(1):gix118. doi: 10.1093/gigascience/gix118 (PMC5765557; doi:10.1093/gigascience/gix118)
Supplement: Reviewer-1-Report-(Original-Submission) -- Tyler Weirick — 20 Jun 2017 Reviewed [file gix118_reviewer-1-report-(original-submission).pdf]

Reviewer Report

Title: circlncRNAnet: An integrated web-based resource for mapping functional networks of long or circular forms of non-coding RNAs

Version: Original Submission **Date:** 6/20/2017

Reviewer name: Tyler Weirick

Reviewer Comments to Author:

Overall the tool implements a wide variety of functionality and appears to function as described. I believe this will be a useful to biologists studying lncRNAs. There are a few areas I believe should be addressed before this paper is ready for publication.1) Quite a large number of sentences seem to be missing citations.Ex:"Both lncRNAs and circRNAs have been found to harbor microRNA response elements (MREs) and potentially act as "miRNA sponges" that sequester these endogenous small RNAs"I guess you are intending for the citations in the following sentence to cover this sentence as well?1) At the moment I believe the writing and formatting is the biggest issue with this paper. These make the article difficult to follow in some sections.1a) The article is verbose, making it difficult to follow.Ex:"At the mechanistic level, regardless of the forms, these non-coding RNA transcripts have been known to impact the expression of messenger RNAs (mRNA) via epigenetic and post-transcriptional regulation."This sentence could be shortened to something like this."Non-coding RNAs are known to affect messenger RNA (mRNA) expression via epigenetic and post-transcriptional regulation"1b) The word usage is a bit strange in some places.Ex:"To circumvent this problem, we have implemented an integrative bioinformatics approach to approach to examine in silico the cellular roles of lncRNAs by mapping their functional networks."Solve is more appropriate instead of circumvent. Circumvent sounds like you are finding a way around the problem without fixing it. Note this sentence is also verbose. I think reducing the verbosity and complexity of the language in the paper will help solve word usage problems.1c) Use more nouns and fewer pronouns.2) Many researchers now consider circRNAs to be lncRNAs. Maybe add something like "… and in fact circRNAs are considered to be a class of lncRNAs by many researchers.. (1)" to "Despite their differences in structure and biosynthesis steps, lncRNAs and circRNAs are much more common in terms of their roles and mechanism in gene regulation"1. Quinn, Jeffrey J., and Howard Y. Chang. "Unique features of long non-coding RNA biogenesis and function." Nature Reviews Genetics 17.1 (2016): 47-62.3) The evidence lncRNAs acting as miRNA sponges is much stronger than the evidence for circRNAs acting as miRNA sponges. I recommend adding a sentence to the effect of "Although the evidence for lncRNA miRNA sponges is much stronger than circRNA sponges (1). " after "Both lncRNAs and circRNAs have been found to harbor microRNA response elements (MREs) and potentially act as "miRNA sponges" that sequester these endogenous small RNAs"1. Militello, Giuseppe, et al. "Screening and validation of lncRNAs and circRNAs as miRNA sponges." Briefings in Bioinformatics (2016): bbw053.2. Boeckel, Jes-Niels, et al. "Identification and characterization of hypoxia-regulated endothelial circular RNA." Circulation research (2015): CIRCRESAHA-115.4) I recommend increasing the quality of the github repository by adding comments to your code and enough documentation that a user could set up their own instance of your server.

Level of Interest

Please indicate how interesting you found the manuscript: An article whose findings are important to those with closely related research interests

Quality of Written English

Please indicate the quality of language in the manuscript: Needs some language corrections before being published

Declaration of Competing Interests

Please complete a declaration of competing interests, considering the following questions:

- Have you in the past five years received reimbursements, fees, funding, or salary from an organisation that may in any way gain or lose financially from the publication of this manuscript, either now or in the future?
- Do you hold any stocks or shares in an organisation that may in any way gain or lose financially from the publication of this manuscript, either now or in the future?
- Do you hold or are you currently applying for any patents relating to the content of the manuscript?
- Have you received reimbursements, fees, funding, or salary from an organization that holds or has applied for patents relating to the content of the manuscript?
- Do you have any other financial competing interests?
- Do you have any non-financial competing interests in relation to this paper?

If you can answer no to all of the above, write 'I declare that I have no competing interests' below. If your reply is yes to any, please give details below.

I declare that I have no competing interests.

I agree to the open peer review policy of the journal. I understand that my name will be included on my report to the authors and, if the manuscript is accepted for publication, my named report including any attachments I upload will be posted on the website along with the authors' responses. I agree for my report to be made available under an Open Access Creative Commons CC-BY license (http://creativecommons.org/licenses/by/4.0/). I understand that any comments which I do not wish to be included in my named report can be included as confidential comments to the editors, which will not be published.

I agree to the open peer review policy of the journal
