# Supplementary material for: circlncRNAnet: an integrated web-based resource for mapping functional networks of long or circular forms of noncoding RNAs
Source: Gigascience. 2017 Nov 29;7(1):gix118. doi: 10.1093/gigascience/gix118 (PMC5765557; doi:10.1093/gigascience/gix118)
Supplement: Reviewer-2-Report-(Original-Submission) -- Wilfried Haerty — 01 Aug 2017 Reviewed [file gix118_reviewer-2-report-(original-submission).pdf]

Reviewer Report

Title: circlncRNAnet: An integrated web-based resource for mapping functional networks of long or circular forms of non-coding RNAs

Version: Original Submission **Date:** 8/1/2017

Reviewer name: Wilfried Haerty

Reviewer Comments to Author:

The pipeline and associated website proposed by the authors is of interest for researchers wanting to better characterize one or several non-coding RNAs.My first major criticism is that the the website was not accessible on July 29th and August 1st which prevented me to assess the proposed tool.The manuscript contains very little detail on how the analyses are performed, I had to check the different scripts available on the GitHub repository was able to find a github repository to understand the analyses performed (differential expression, co-expression analysis). Additionally there is no list of the packages called by the program, nor does the manuscript highlight the species and references supported. Furthermore there is no information regarding the parameters used to run the different programs (miRanda, RNAHybrid ...). The authors need to significantly develop this, it is nice to have examples of the different analyses the pipeline enables but it is of utmost importance that the reader gains a good grasp of the tools and analyses performed by the pipeline and the parameters used.Minor comments the authors mention that the users can define the gene list after differential expression using the fold changes and p-values. Are they using the p-value or the adjust p-value? From the scripts available on github (deseq2_annotation.r) it appears that the column used is the uncorrected p-value which implies a list of differentially expressed genes heavily contaminated by false positives.It would be very useful to the user if the authors could guide them in the choice of the Pearson's coefficient of correlation, such as using a randomization approach on the submitted sets to identify the thresholds.

Level of Interest

Please indicate how interesting you found the manuscript: An article of limited interest

Quality of Written English

Please indicate the quality of language in the manuscript: Acceptable

Declaration of Competing Interests

Please complete a declaration of competing interests, considering the following questions:

- Have you in the past five years received reimbursements, fees, funding, or salary from an organisation that may in any way gain or lose financially from the publication of this manuscript, either now or in the future?

- Do you hold any stocks or shares in an organisation that may in any way gain or lose financially from the publication of this manuscript, either now or in the future?
- Do you hold or are you currently applying for any patents relating to the content of the manuscript?
- Have you received reimbursements, fees, funding, or salary from an organization that holds or has applied for patents relating to the content of the manuscript?
- Do you have any other financial competing interests?
- Do you have any non-financial competing interests in relation to this paper?

If you can answer no to all of the above, write 'I declare that I have no competing interests' below. If your reply is yes to any, please give details below.

I have no competing interest

I agree to the open peer review policy of the journal. I understand that my name will be included on my report to the authors and, if the manuscript is accepted for publication, my named report including any attachments I upload will be posted on the website along with the authors' responses. I agree for my report to be made available under an Open Access Creative Commons CC-BY license (http://creativecommons.org/licenses/by/4.0/). I understand that any comments which I do not wish to be included in my named report can be included as confidential comments to the editors, which will not be published.

I agree to the open peer review policy of the journal
